# Design and development of stapled transmembrane peptides that disrupt the activity of G-protein–coupled receptor oligomers

**DOI:** 10.1074/jbc.RA119.009160

**Published:** 2019-08-29

**Authors:** Joaquín Botta, Lucka Bibic, Patrick Killoran, Peter J. McCormick, Lesley A. Howell

**Affiliations:** ‡ Centre for Endocrinology, William Harvey Research Institute, Bart's and the London School of Medicine and Dentistry, Queen Mary University of London, Charterhouse Square, London EC1M 6BQ, United Kingdom; § School of Pharmacy, University of East Anglia, Norwich Research Park, Norwich NR4 7TJ, United Kingdom; ¶ School of Pharmacy and Biomolecular Sciences, Liverpool John Moores University, James Parsons Building, Byrom Street, Liverpool L3 3AF, United Kingdom; ‖ School of Biological and Chemical Sciences, Queen Mary University of London, Mile End Road, London E1 4NS, United Kingdom

**Keywords:** G protein-coupled receptor (GPCR), cannabinoid receptor type 1 (CB1), dimerization, cell-penetrating peptide (CPP), peptide chemical synthesis, protein-protein interaction, serotonin receptor type 2A (5HT2A), NanoLuc binary technology (NanoBiT), hydrocarbon stapling, transmembrane peptide, bimolecular fluorescence complementation

## Abstract

Membrane proteins can associate into larger complexes. Examples include receptor tyrosine complexes, ion channels, transporters, and G protein–coupled receptors (GPCRs). For the latter, there is abundant evidence indicating that GPCRs assemble into complexes, through both homo- and heterodimerization. However, the tools for studying and disrupting these complexes, GPCR or otherwise, are limited. Here, we have developed stabilized interference peptides for this purpose. We have previously reported that tetrahydrocannabinol-mediated cognitive impairment arises from homo- or heterooligomerization between the GPCRs cannabinoid receptor type 1 (CB_1_R) and 5-hydroxytryptamine 2A (5-HT_2A_R) receptors. Here, to disrupt this interaction through targeting CB_1_–5-HT_2A_ receptor heteromers in HEK293 cells and using an array of biochemical techniques, including calcium and cAMP measurements, bimolecular fluorescence complementation assays, and CD-based helicity assessments, we developed a NanoLuc binary technology (NanoBiT)-based reporter assay to screen a small library of aryl-carbon–stapled transmembrane-mimicking peptides produced by solid-phase peptide synthesis. We found that these stapling peptides have increased α-helicity and improved proteolytic resistance without any loss of disrupting activity *in vitro*, suggesting that this approach may also have utility *in vivo*. In summary, our results provide proof of concept for using NanoBiT to study membrane protein complexes and for stabilizing disrupting peptides to target such membrane complexes through hydrocarbon-mediated stapling. We propose that these peptides could be developed to target previously undruggable GPCR heteromers.

## Introduction

The discovery that G protein–coupled receptors (GPCRs)^3^ could oligomerize, termed homo- or heterodimerization, sparked an intense debate that has moved on from whether they can oligomerize to why, how, and how frequently they oligomerize and how we might target oligomers for therapeutic purposes. Several X-ray–resolved GPCR crystal structures have revealed common dimeric interfaces stabilizing oligomeric arrangements within the rhodopsin-like family receptors. Dimers having an interface involving the transmembrane (TM) domains TM1, TM2, and H8 appear to be a commonly conserved organization, including the structures of the rhodopsin, opsin, metarhodopsin II, μ- and κ-opioid, and β1 adrenergic receptors (1–6). An additional interface involving the TM4 and TM5 domains was also shown in the squid rhodopsin and the β_1_-adrenergic receptors (2, 7). Furthermore, the crystal structures of the CXCR4 and μ-opioid receptors revealed a substantial buried surface area of 850 and 1,492 Å^2^, respectively, comprised of the TM5 and TM6 domains (3, 8). Selective disruption of dimers using synthetic peptides harboring the same amino acid sequence as the interacting TM domains has helped to validate and understand the functional consequences of receptor oligomerization, including the β_2_-adrenergic, CXCR4, oxytocin, and apelin receptor homooligomers (9–12). Synthetic peptides have also provided unique tools to map the interfaces and understand the biological relevance of class A GPCR heteromerization (13, 14). An additional advantage of TM peptides is that, unlike in knockout studies or deletions, the peptides preserve the functional single protomers and allow one to discriminate between those effects driven by the interacting receptors and those derived from the individual protomers. An example of the former is the *in vivo* disruption of the cross-class angiotensin receptor subtype 1a (AT1aR) and secretin receptor heteromers with a TM1 AT1aR mimetic peptide, reducing hyperosmolality-induced drinking behavior (13).

In cases where heteromer disruption might serve a therapeutic purpose, it would be advantageous to translate TM peptides not only as tools, but into druglike entities. Peptides in general are considered poor druglike molecules, although this view is changing. Efficacy is often compromised *in vivo* due to a loss of secondary structure; cellular uptake is poor, and finally peptides are highly susceptible to proteolysis. Stapling of the peptide backbone can help to overcome these limitations; the bioactive conformation of the peptide is maintained, and careful positioning and choice of staple can result in a high-affinity binder with improved cellular uptake and stability. Stapled peptides therefore represent an attractive approach to developing more druglike peptides. For a recent review on stapled peptides, see Ali *et al.* (15). We have shown previously that the undesired effect of cognitive impairment in the presence of *trans*-Δ9-tetrahydrocannabinol (THC) is driven by homo-/heterooligomerization between CB_1_ and 5-HT_2A_ receptors (16). To disrupt this interaction, we developed a NanoLuc binary technology (NanoBiT)-based assay for the screening of a small library of aryl-hydrocarbon–stapled CB_1_R TM5-mimicking peptides to target CB_1_–5-HT_2A_ receptor heteromers. NanoBiT is a luciferase-based complementation assay designed to interrogate protein–protein interactions in live cells (17). Using this approach, we found that stapling peptides led to increased α-helicity and improved proteolytic resistance without any loss of function, suggesting that this approach may improve these molecules chances *in vivo*.

## Results

### Establishing the NanoBiT system for heteromer-screening purposes

The CB_1_ and 5-HT_2A_ receptor heteromer has been recently characterized both *in vivo* and in heterologous expression systems using a broad range of biochemical approaches (16). In the case of these heteromers, the cognitive impairment induced by THC is abrogated after treatment with CB_1_R TM5 peptides while maintaining its antinociceptive properties (16). Therefore, for therapeutic purposes, there needs to be prevention of heteromer formation but preservation of the individual protomer's function. Thus, we first sought to develop a NanoBiT-based assay for the screening of a small library of hydrocarbon-stapled CB_1_R TM5-mimicking peptides. To assess whether NanoBiT BiLC may be a suitable system to study GPCR oligomerization, we generated CB_1_R and 5-HT_2A_R constructs with the small and large BiT pairs (SmBiT and LgBiT, respectively) attached to the C terminus end of both receptors. A total of four fusion proteins were generated, with Sm/LgBiT fused after the Gly/Ser-rich flexible linker and under the control of the herpes simplex virus thymidine kinase gene promoter (HSV-TK) (Fig. S1*A*). Next, we performed conformational screenings to assess the optimal configuration for all receptor pairs. Accordingly, when analyzing CB_1_R–5-HT_2A_R heteromers, HEK293 cells were transiently transfected with all possible combinations of 5-HT_2A_R Lg/SmBiT and CB_1_R Lg/SmBiT at two different DNA ratios (Fig. S1*B*). Surprisingly, none of the analyzed configurations yielded a positive interaction. Similarly, when addressing the formation of CB_1_R and 5-HT_2A_R homodimers, none of the examined orientations or DNA ratios exhibited significant differences compared with the individual receptors when expressed by themselves (Fig. S1, *C* and *D*). Importantly, to rule out whether these negative results might reflect the unsuitability of the NanoBiT system for the analysis of GPCR oligomerization rather than any kind of experimental hindrance, we analyzed the known interaction between the protein kinase A catalytic (PRKACA) and type 2A regulatory (PRKAR2A) subunits. This protein pair positive control has been previously optimized, with LgBiT-PRKAR2A and SmBiT-PRKACA the optimal configuration (17). In agreement, co-transfection of both proteins resulted in a significant increase in the luminescence recorded over the different receptor ratios. Furthermore, co-transfection of LgBiT-PRKAR2A with a noninteracting fusion protein (HaloTag®-SmBiT) did not yield any increase in luminescence (Fig. S1*E*), supporting the specificity of the detected interaction and the suitability of this system under our assay conditions. However, our results do not reveal if the complementary fusions restrict 5-HT_2A_R or CB_1_R functionality or if there is indeed a lack of complementation. Importantly, it should be noted that to minimize potential nonspecific interactions, all generated constructs were under the control of the HSV-TK promoter.

Thus, to address the lack of a BiLC signal, we performed secondary messenger signaling experiments and recloned all four receptor configurations under the control of the high-level expression cytomegalovirus (CMV) promoter. Interestingly, all 5-HT_2A_R constructs in an HSV-TK context failed to elicit intracellular calcium release (canonical signaling pathway downstream to the G_q/11_- coupled 5-HT_2A_R) after agonist stimulation (18). However, both 5-HT_2A_R LgBiT and 5-HT_2A_R SmBiT displayed similar efficacies and potencies compared with the WT receptor when expressed under the control of the CMV promoter (Fig. S2*A*). Similarly, we assessed CB_1_R-driven adenylate cyclase inhibition with analogous results. Both CMV-regulated CB_1_R constructs inhibited the forskolin-induced cAMP release with equivalent potencies and efficacies to the WT CB_1_R receptor. However, under the HVS-TK promoter, CB_1_R LgBiT failed to signal through heterotrimeric G_i/o_ proteins, and CB_1_R SmBiT exhibited a reduced adenylate cyclase inhibitory activity (∼20%) when compared with WT CB_1_R receptor (Fig. S2*B*). These results indicate that the NanoBiT fusions do not adversely affect 5-HT_2A_ or CB_1_ receptor functionality, as the ligand potencies and maximal efficacies are within the WT receptor ranges. Thus, the absence of luciferase complementation between HSV-TK–regulated constructs most likely reflects low levels of receptor expression due to the weaker promoter rather than steric hindrance of the interacting pairs (19).

### NanoBiT can be used to estimate receptor affinities

**Figure 1.**
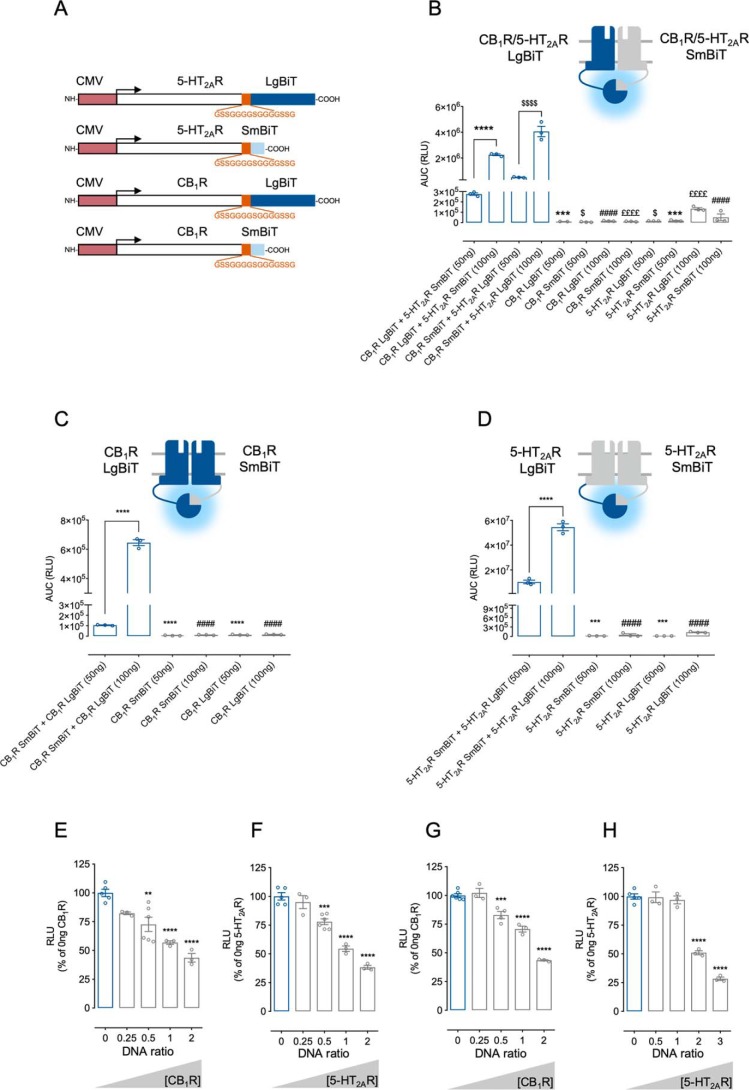
**NanoBiT complementation can be used to estimate oligomer affinities.** *A*, *schematic representation* of the CMV-promoter NanoBiT fusion proteins. HEK293 cells were transiently transfected with all possible orientations of LgBiT and SmBiT C-terminal fusions at two different DNA ratios (50 or 100 ng of receptor/well) to assess 5-HT_2A_R–CB_1_R heteromers (*B*) or CB_1_R (*C*) and 5-HT_2A_R (*D*) homomers. Disruption of CB_1_R (*E*) and 5-HT_2A_R (*F*) homomers and 5-HT_2A_R–CB_1_R heteromers (*G* and *H*) was assessed in the presence of increasing untagged receptor concentrations (see *below panels*). In *B–D*, data are mean area under the curve (RLU) ± S.E. (*n* = 3). In *B*, statistical significance was evaluated by one-way analysis of variance (ANOVA) followed by Bonferroni post hoc tests showing significant effects for CB_1_R LgBiT + 5-HT_2A_R SmBiT (50 ng) against the same configuration at 100 ng/well or each equivalent individual construct (***, *p* ≤ 0.001; ****, *p* ≤ 0.0001), for CB_1_R LgBiT + 5-HT_2A_R SmBiT (100 ng) over each equivalent individual construct (####, *p* ≤ 0.0001), for CB_1_R SmBiT + 5-HT_2A_R LgBiT (50 ng) over the same configuration at 100 ng/well or each equivalent individual construct ($, *p* ≤ 0.05; $$$$, *p* ≤ 0.0001), and for CB_1_R SmBiT + 5-HT_2A_R LgBiT (100 ng) over each equivalent individual construct (££££, *p* ≤ 0.0001). In *C* and *D*, statistical significance was evaluated as in *B*, showing significant effects for CB_1_ SmBiT + CB_1_R LgBiT (50 ng) or 5-HT_2A_R SmBiT + 5-HT_2A_R LgBiT (50 ng) over the same configuration at 100 ng/well or each equivalent individual construct (***, *p* ≤ 0.001; ****, *p* ≤ 0.0001) and for CB_1_ SmBiT + CB_1_R LgBiT (100 ng) or 5-HT_2A_R SmBiT + 5-HT_2A_R LgBiT (100 ng) over each individual construct (####, *p* ≤ 0.0001). In *E–H*, values are mean ± S.E. (*error bars*) (*n* ≥ 3) of the percentage of luminescence normalized to 0 ng of nontagged competitor. For each condition, statistical significance was evaluated by one-way ANOVA followed by Bonferroni's post hoc tests showing significant effects over 0 ng of nontagged competitor (**, *p* ≤ 0.01; ***, *p* ≤ 0.001; ****, *p* ≤ 0.001). *CMV*, human cytomegalovirus immediate-early promoter.

Accordingly, as in the previous studies illustrated in Fig. 1*A*, we repeated the conformational screenings with the new set of constructs under the CMV promoter. When analyzing 5-HT_2A_R–CB_1_R heteromerization, we detected a significant increase in the luminescence for all receptor combinations, with 5-HT_2A_R LgBiT:CB_1_R SmBiT being the optimal pair in terms of assay window (Fig. 1*B*). To validate the specificity of the interaction, increasing amounts of untagged 5-HT_2A_R and CB_1_R receptors were transfected in the presence of a fixed 5-HT_2A_R LgBiT:CB_1_R SmBiT ratio. Accordingly, we observed a decreased luminescence with increasing levels of both untagged proteins (Fig. 1, *E* and *F*), indicating that the detected interaction was not driven by the finite affinity between the NanoLuc subunits. In parallel, we assessed CB_1_R and 5-HT_2A_R homomerization with similar results. Co-transfection of both CB_1_R-interacting pairs resulted in a significant increase in luminescence that could be reverted by increasing untagged CB_1_R concentrations (Fig. 1, *C* and *G*). Similarly, the specific interaction between 5-HT_2A_R LgBiT and 5-HT_2A_R SmBiT was hindered when titrating increasing concentrations of untagged 5-HT_2A_R (Fig. 1, *D* and *H*). Importantly, when comparing the relative intensities of these interactions, we found that 5-HT_2A_R homodimers displayed the highest luminescent signals, followed by 5-HT_2A_R–CB_1_R heteromers and CB_1_R homodimers (RLU for 5-HT_2A_R:5-HT_2A_R > 5-HT_2A_R:CB_1_R > CB_1_R:CB_1_R). In addition, higher nontagged 5-HT_2A_R concentrations were necessary to displace 5-HT_2A_R homodimers (Fig. 1*H*). However, our results cannot discriminate whether it reflects the relative affinities between the interacting receptors or a more proximal distance of the NanoBiT pairs in the C terminus of the receptors. Altogether, our data strongly support NanoBiT BiLC as a nondestructive and powerful tool to study GPCRs oligomerization, providing a specific and sensitive assay to detect these receptor complexes in live cells.

### Comparison of NanoBiT with Venus bimolecular fluorescent complementation

**Figure 2.**
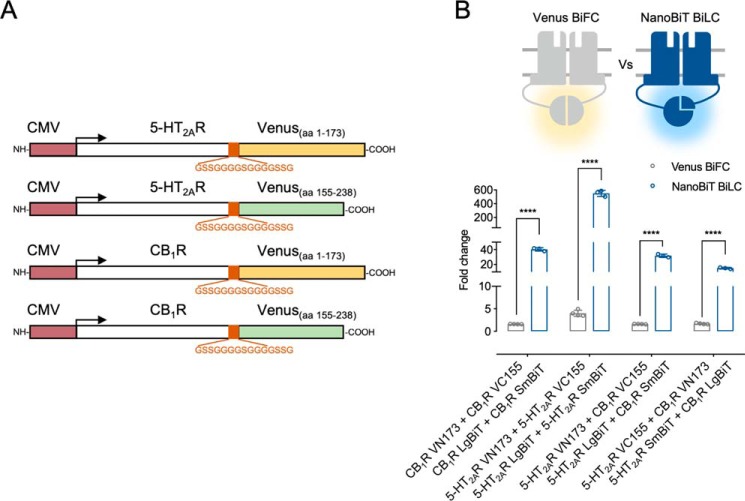
**Comparative analysis of NanoBiT with Venus BiFC.** *A*, *schematic representation* of the CMV-driven Venus YFP BiFC fusion proteins. In *B*, HEK293 cells were transiently transfected with all possible Venus YFP complementary orientations and compared with its equivalent NanoBiT BiLC pairs to assess CB_1_R and 5-HT_2A_R homomers and 5-HT_2A_R–CB_1_R heteromers. Data are mean ± S.E. (*error bars*) (*n* ≥ 3) of -fold change, calculated as the ratio between each condition and the individual receptor construct with the highest luminescence/fluorescence values. Statistical significance was evaluated by unpaired *t* tests between groups followed by Holk–Sidak corrections for multiple comparison (****, *p* ≤ 0.0001). *CMV*, human cytomegalovirus immediate-early promoter. *aa*, amino acids.

To obtain a more comprehensive understanding of bimolecular complementation assays to study GPCR oligomerization, we sought to address whether NanoBiT could provide better results compared with bimolecular fluorescent complementation. To this end, we developed a Venus bimolecular fluorescent complementation (BiFC) assay to study CB_1_R and 5-HT_2A_R homo-/heteromerization. Specifically, fragments derived from the truncated Venus fluorescent protein at either position Asp-155 (VC155; amino acid residues 155–238) or Asp-173 (VN173; amino acid residues 1–173) were fused after the Gly/Ser-rich flexible linker to the C terminus of both CB_1_ and 5-HT_2A_ receptors (Fig. 2*A*). This strategy has been extensively applied to the study of protein–protein interactions (PPIs) and takes advantage of Venus, a variant of the enhanced yellow fluorescent protein (YFP) with improved sensitivity to chromophore maturation under physiological temperatures (21). Several groups have used this approach to study GPCR oligomerization, including heteromers between the adenosine A_2A_ and the dopamine D_2_ receptors, dopamine D_2_ oligomers, or neuropeptide Y Y_1_/Y_5_ receptor heterodimers (19–21).

Attachment of both Venus hemiprotein fragments to the 5-HT_2A_R C-terminal tail (5-HT_2A_R–VN173 and 5-HT_2AR_–VC155 constructs) did not impact receptor function, with equivalent [Ca^2+^]*_i_* release dose–response curves compared with the WT receptor (Fig. S3*A*). Similarly, the VC155 fragments fused to CB_1_R (CB_1_R-VC155) did not affect CB_1_R-mediated cAMP release inhibition. However, although its maximal efficacy remained unaltered, VN1733 fusion to CB_1_R (CB_1_R-VN173) resulted in ∼10-fold reduction in WIN 55212-2 (*WIN*) potency (Fig. S3*B*). Next, we proceeded to compare both protein complementation assays. For CB_1_R and 5-HT_2A_R homomers, BiFC experiments were performed under the same conditions that yielded the optimal assay windows in the NanoBiT BiLC experiments (Fig. 2). When assessing 5-HT_2A_R–CB_1_R heteromers, both possible receptor configurations (5-HT_2A_R–VN173:CB_1_R–VC155 and 5-HT_2A_R–VC155:CB_1_R–VN173) were taken into account. Surprisingly, 24 and 48 h after reverse transfection, none of the analyzed BiFC combinations yielded significant fluorescent levels (data not shown), suggesting time-dependent protein maturation and/or folding. Therefore, the following BiFC experiments were performed 48 h post-transfection (see “Experimental procedures”), although BiLC assays remained under the same setup (24 h post-transfection). Compared with Venus BiFC, NanoBiT complementation provided higher assay windows over all of the oligomeric configurations (Fig. 2). Specifically, we observed a 20-fold increase for CB_1_R homodimers, 130-fold increase for 5-HT_2A_R homodimers, and 9–18-fold increase for 5-HT_2A_R–CB_1_R heteromers. Interestingly, the relative fluorescent/luminescent intensities for the different receptor pairs followed the same trend across both methods (RFU/RLU for 5-HT_2A_R:5-HT_2A_R > 5-HT_2A_R:CB_1_R > CB_1_R:CB_1_R), suggesting that this could reflect the affinity between these oligomeric arrangements.

### N-terminal GPCR fusions are also functional for NanoBiT complementation

**Figure 3.**
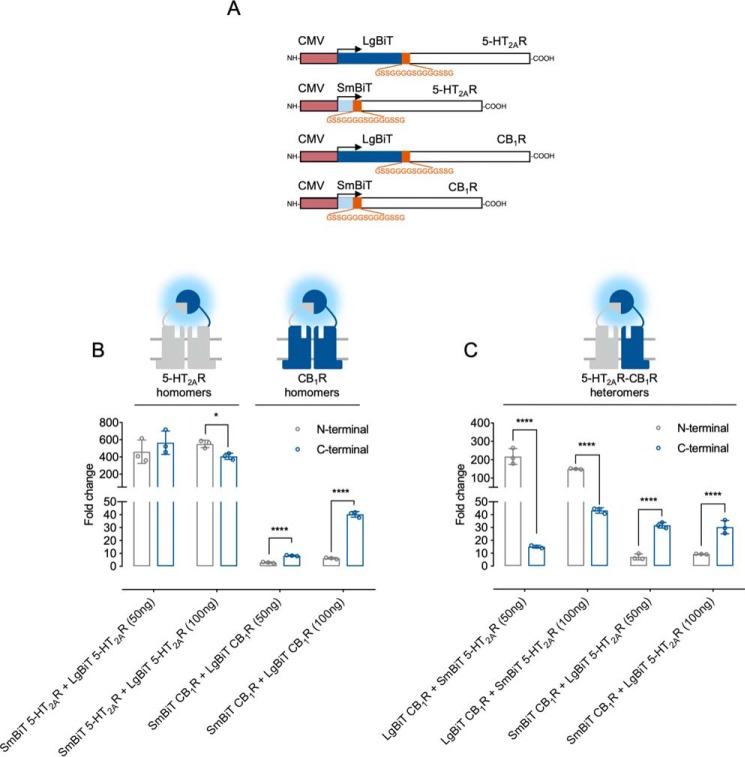
**Assay optimization for the screening of peptides disrupting 5-HT_2A_R–CB_1_R heteromers.** *A*, *schematic representation* of the CMV-driven NanoBiT N-terminally tagged CB_1_ and 5-HT_2A_ receptors. Shown is a comparison between N-terminal or C-terminal tagging to assess CB_1_R and 5-HT_2A_ receptor homomers (*B*) or 5-HT_2A_R–CB_1_R heteromers (*C*). Data are mean ± S.E. (*error bars*) (*n* = 3) of -fold change, calculated as the ratio between each condition and the individual receptor construct with the highest luminescence background. Statistical significance was evaluated by unpaired *t* tests between groups followed by Holk–Sidak corrections for multiple comparison (*, *p* ≤ 0.05; ****, *p* ≤ 0.0001). *CMV*, human cytomegalovirus immediate-early promoter.

Next, we sought to test whether N-terminal tagging might yield better assay windows (Fig. 3*A*). SmBiT and LgBiT fusion to the 5-HT_2A_R extracellular end did not impact function, as treatment with the agonist (±)-2,5-dimethoxy-4-iodoamphetamine hydrochloride (DOI) induced maximal calcium release, although a small reduction in DOI potency was observed in the LgBiT–5-HT_2A_R construct (Fig. S4*A*). The homologous CB_1_R constructs remained unaltered, with virtually the exact potencies and maximal responses as the WT receptor (Fig. S4*B*). Next, we compared N- or C-terminal NanoBiT-tagged receptors, accounting for their ability to reveal CB_1_R and 5-HT_2A_R homo-/heteromers. When measuring the interaction between receptors from the same type, NanoBiT attachment to the C-terminal domain provided the optimal orientation for CB_1_R homomers (Fig. 3*B*). In the case of 5-HT_2A_R homomers, N-terminally tagging resulted in a discrete but significant improvement in the assay window at high DNA concentrations (Fig. 3*B*). Interestingly, the biggest difference was observed when assessing the optimal 5-HT_2A_R–CB_1_R heteromeric conformation, with the N-terminal fusions providing the best results (Fig. 3*C*). Importantly, a ∼200-fold increase was achieved at low DNA-transfected concentrations, therefore reducing the probability of stochastic nonspecific interactions and thus more closely mimicking physiological expression levels. The LgBiT–CB_1_R and SmBiT–5-HT_2A_R combination was therefore used in all further experiments.

### Design and synthesis of stapled peptides

In previous studies, we demonstrated that 5-HT_2A_R–CB_1_R heteromers could be selectively disrupted using synthetic peptides mimicking the CB_1_R TM5 and TM6 domains (16), fused to the HIV-TAT (GRKKRRQRRR) cell-penetrating amino acid sequence (CPS) (23). In the same study, we also showed that the peptide mimicking CB_1_R TM7 did not disrupt the heteromer. As part of our preliminary work, we had previously identified a truncated CB_1_R TM5 amino acid sequence fused to TAT (VYAYMYILWGRKKRRQRRR) capable of disrupting the 5-HT_2A_R–CB_1_R heteromer.^4^ We therefore chose to design stapled peptides based on the amino acid sequences of the truncated CB_1_R TM5 to aid with the synthesis and purification of the peptides as well as solubility. In addition, amino acid sequences of 20–25 amino acid residues or less are generally recommended for stapling (24).

**Figure 4.**
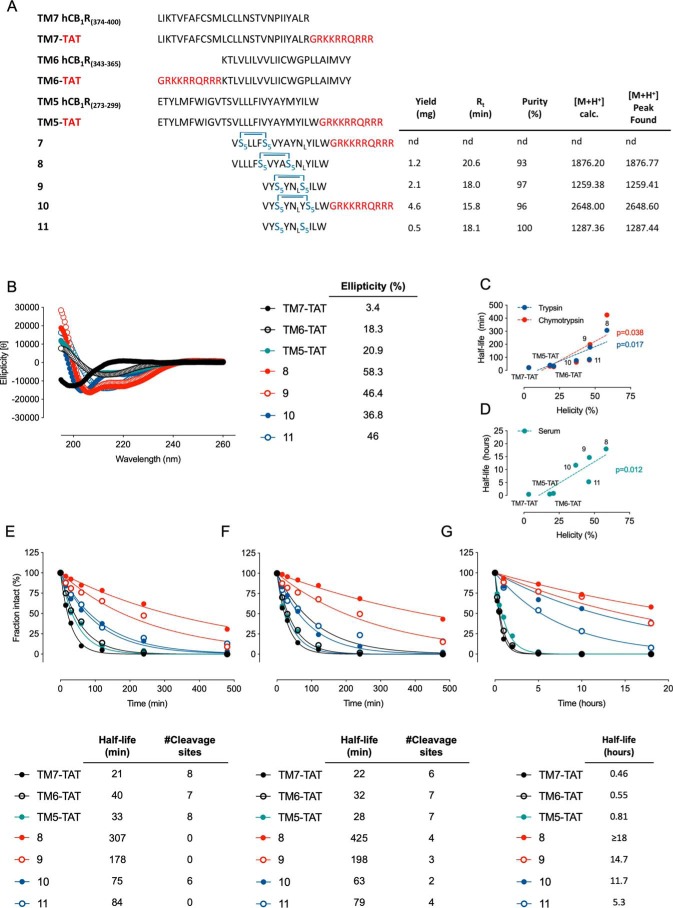
**Biophysical analysis of stapled peptides.** *A*, amino acid sequence alignment of the targeted TM regions and disrupting peptides. Cys residues are in the reduced state. *Blue bridges* indicate the stapled amino acid residues. The HIV-TAT amino acid sequence is displayed in *red*. Yields, purity, and MALDI-TOF data are shown for the stapled peptides and negative control. *B*, CD analysis of 30–50 μm peptides at 20 °C. For proteolytic stability studies, the peptide solution (55.5 μm for trypsin and chymotrypsin and 11.1 μm for serum) was incubated in the presence of 0.55 μg/ml trypsin from porcine pancreas (*E*), α-chymotrypsin (*F*), or mouse serum (*G*) at 37 °C for the indicated times (indicated on the *x axis*). Data are mean ± S.E. (*error bars*) (*n* = 3) percentage of intact peptide normalized to *t* = 0. Proteolytic half-lives and putative cleavage sites (predicted using the Expasy bioinformatics server's model with a 50% probability of cleavage) are indicated at the *bottom* of each *panel*. Positive correlation between helicity and half-life in trypsin/chymotrypsin (*C*) or serum (*D*) was determined by two-tailed Pearson's correlation test.

Accordingly, using these structures as a starting point, we hypothesized that shortening their length, in combination with hydrocarbon peptide stapling, could result in peptides with more druglike properties (Fig. 4*A*). This strategy, through the incorporation of α-methyl-α-alkenyl amino acids, combines the methylation of the α-carbon atom together with the introduction of a covalent side chain–to–side chain cross-link, resulting in peptides with increased α-helicity and improved proteolytic resistance (25, 26). We next evaluated where to add the hydrocarbon staples. One major consideration is charge. An overall positive net charge is favorable after staple installation to aid with cellular uptake. The location of the positive amino acids is also influential and if possible should be located at the C terminus. Molecular modeling identified potential sites within these TM5 peptide amino acid sequences to introduce the hydrocarbon staple (Table 1). The positions (facing the membrane of CB_1_R) to build the stapled peptides (at *i* and *i* + ¾) are shown in *red*. The TAT amino acid sequence is shown in *orange*.

**Table 1:**
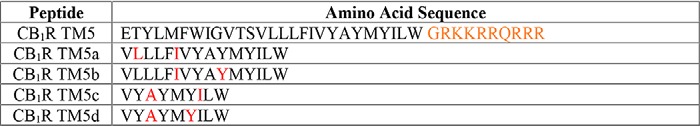
**Potential sites within the CB_1_R TM5 peptide amino acid sequence to introduce the hydrocarbon staple were identified based on a previously published molecular model (see “Experimental procedures”)** The positions (facing the lipids) to build the stapled peptides (at *i* and *i* + 34) are shown in *red.* The TAT amino acid sequence is shown in *orange*.

**Scheme 1.**
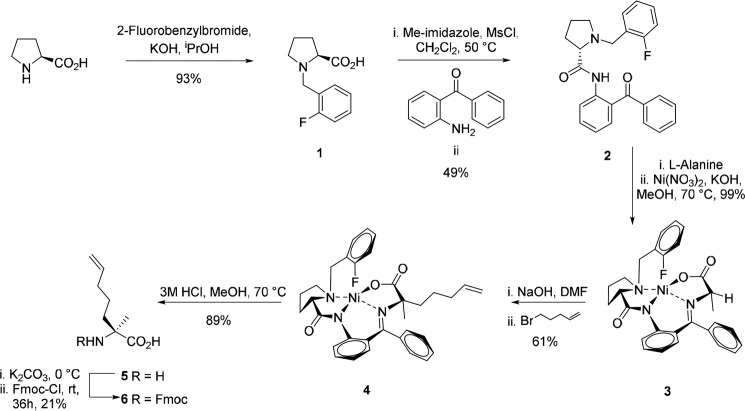
**Synthesis of the Fmoc-protected unnatural amino acid *S*-pentenylalanine (Fmoc–S_5_-OH)**.

Peptides are often thought of as poor drug molecules, as 1) *in vivo* their efficacy is compromised due to a loss of secondary structure, 2) they often have poor cellular uptake, and 3) they are highly susceptible to proteolysis. Stapling of the peptide backbone is an attractive strategy to overcome these limitations; the bioactive conformation of the peptide is maintained, and careful positioning and choice of staple can result in a high-affinity binder with improved cellular uptake and stability. Next is the choice of staple to use. We chose to incorporate the all-hydrocarbon staple for α-helical peptide stabilization. This was first reported in 2000 by Schafmeister *et al.* (27) and has since found use in a variety of applications, including cancer, infectious diseases, and neuroscience (28). In addition, this staple has been shown to protect the peptide against proteolysis as the vulnerable peptide bonds are sequestered in the interior of the helix. The building block for single-turn hydrocarbon stapling is the unnatural amino acid *S*-pentenylalanine (Fmoc–S_5_-OH). Although available commercially, we chose to synthesize the Fmoc-protected version in-house, adapting methods reported by Jamieson and Ryzhov (Scheme 1) (29, 30). Briefly, *N*-alkylation of proline with 2-fluorobenzyl bromide gave **1** in almost quantitative yields. This was followed by the condensation of **1** with 2-aminobenzophenone to give the chiral auxiliary (*S*)-*N*-(2-benzoylphenyl)1(2-fluorobenzyl)-pyrrolidine-2-carboxamide **2** (2-FBPB). Complexation with nickel nitrate and l-alanine gave (*S*)-Ni-Ala-2FBPB **3** in almost quantitative yields. This was followed by asymmetric alkylation to yield complex **4** in a good yield, which was subsequently decomposed under acidic conditions to give *S*-pentenylalanine **5**. Finally, this was Fmoc-protected by reaction with Fmoc chloride under basic conditions to afford Fmoc–*S*-pentenylalanine **6**. Following synthesis of the unnatural amino acid, we turned our attention to the synthesis of the CB_1_R TM5 peptides.

**Scheme 2.**
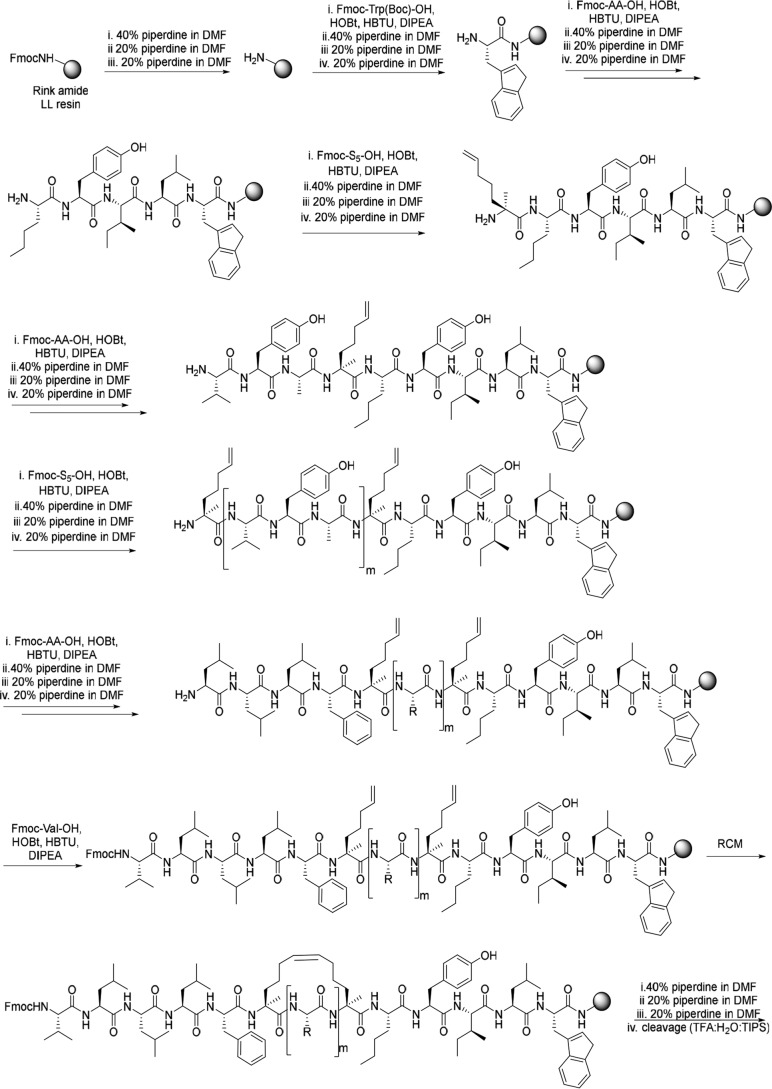
**Synthesis of stapled peptide 8**.

Five peptides (**7–11**) were designed for our study, including one negative control (**11**) (Fig. 4*A*). Methionine amino acid residues were replaced with norleucine amino acid residues to avoid any complications or unwanted side reactions during ring closing metathesis reactions. Peptides were synthesized using Fmoc-based solid-phase peptide synthesis using procedures reported previously (9). Scheme 2 outlines an example synthesis. During assembly, the Fmoc–S_5_-OH is incorporated into positions separated by 2 or 3 amino acid residues, as shown in Fig. 4*A*. Coupling times were increased from 45 min to 60 min for the Fmoc–S_5_-OH and for the residue following the olefinic unnatural amino acid. The assembled peptides (**7–10**) were then subjected to the ring-closing metathesis (RCM) reaction while still on the solid support. This was monitored using reversed-phase HPLC (RP-HPLC); note that peptide **7** did not undergo the RCM reaction. Following a final Fmoc deprotection step, the stapled peptides were cleaved from the solid support under acidic conditions and subsequently purified using preparative RP-HPLC and freeze-dried from water as described previously (30). The negative control **11** was treated in an identical fashion with the exception that the RCM step was omitted. Purified peptides were analyzed using analytical RP-HPLC to assess purity and MALDI-TOF MS to determine accurate mass. All peptides were soluble in water/aqueous buffer.

The administration of peptides to disrupt GPCR interactions is relatively new, with no available information regarding their pharmacokinetics and/or pharmacodynamics. Often with peptides, the *in vivo* efficacy is compromised due to a loss of secondary structure; to assess the helicity of the peptides and whether incorporation of the staple had increased the helical nature of the peptides, we employed CD spectroscopy (Fig. 4*B*). CB1 TM5-TAT, TM6-TAT, and TM7-TAT all displayed relatively low helicity in solution (less than ∼20%). Incorporation of the staples increased the helical nature of the stapled peptides **8–10** in all cases when compared with the TM5-TAT peptide. Peptide **8** demonstrated the highest helicity of 58.3%. Interestingly, the negative control **11**, where the olefinic amino acids are present but not stapled, displayed an almost identical helicity (46.0%) when compared with the stapled version **9**. When TAT is added to the amino acid sequence **10**, the helicity drops slightly to 36.8%. A second challenge facing protein/peptide therapies is proteolytic degradation. Consequently, we subjected our peptide library to *in vitro* trypsin, chymotrypsin, and serum proteolytic stability. When monitoring trypsin (0.55 μg/ml) degradation kinetics (Fig. 4*E*), a rapid proteolysis was observed for the TM7-TAT, TM6-TAT, and TM5-TAT control peptides (55 μm each), with half-lives ranging from 20 to 40 min. The short-stapled CB_1_R TM5 amino acid sequence harboring the TAT amino acid sequence **10** displayed a longer half-life, with a 2–3-fold enhancement in trypsin resistance. Furthermore, removal of the TAT amino acid sequence in the stapled peptides (**8** and **9**) yielded the longest half-lives (∼5 h), as neither lysine nor arginine amino acid residues were available for the trypsin to cleave. This increase in half-life was also observed for the negative controls where the TAT amino acid sequence was removed. Chymotrypsin proteolytic kinetics showed similar results (Fig. 4*F*); the full-length TM7-TAT, TM6-TAT, and TM5-TAT peptides were more susceptible to cleavage (half-lives ranging from 20 to 30 min). Again, a ∼2-fold resistance improvement was detected for stapled peptide **10** bearing the TAT amino acid sequence. Likewise, **8** and **9** were the peptides with longer half-lives (5–6 h) (Fig. 4*F*). In mouse serum, a more physiologically relevant context, the TM7-TAT, TM6-TAT, and TM5-TAT peptides were rapidly degraded, with a 1-h incubation sufficient to break down 50% of them (Fig. 4*G*). Hydrocarbon stapling translated to an even higher serum stability compared with trypsin and chymotrypsin, with peptides **8** and **9** displaying half-lives of more than 10 h. These results positively correlate with the respective helicity of each peptide, as the reinforcement of α-helical structure limits the peptides to adopt the extended conformation required by proteases to hydrolyze the amide bonds (25). Accordingly, we observed a strong positive correlation between helicity and proteolytic resistance (*r* = 0.84, 0.78, and 0.87 for trypsin, chymotrypsin, and mouse serum, respectively; Fig. 4, *C* and *D*). Finally, we investigated the effects of the peptides on cell proliferation and toxicity using a label-free assay. Specifically, we analyzed cell viability based on changes in electrical impedance over time. No statistically significant reduction in viability of the cells was observed over a 24- or 48-h period (Fig. S5).

### Stapled peptides are effective at disrupting heteromers

**Figure 5.**
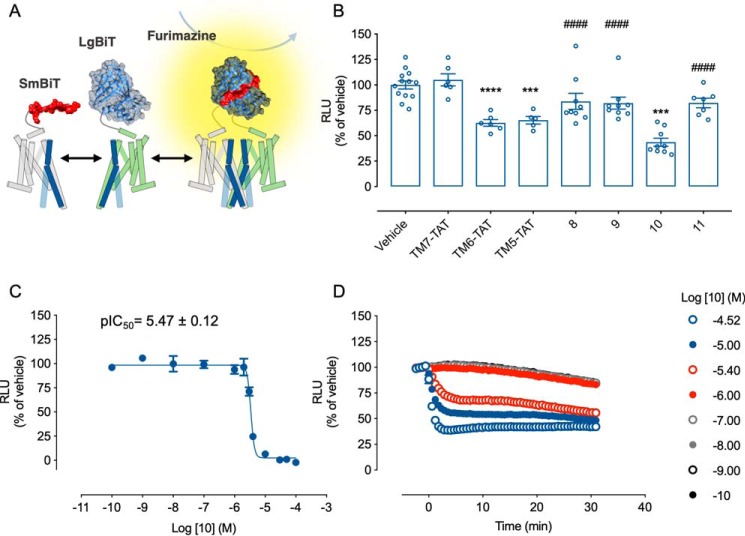
**Identification of a small-stapled TM peptide disrupting 5-HT_2A_R–CB_1_R heteromers.** *A*, *schematic representation* of the NanoBiT assay using N-terminally labeled constructs. In *B*, HEK293 cells transiently expressing the LgBT–CB_1_R and SmBiT–5-HT_2A_R complementary pairs were preincubated for 1 h at 37 °C with the indicated peptides (4 μm) or vehicle prior to luminescence recording. Data are mean RLU ± S.E. (*error bars*) (*n* ≥ 5) percentage of luminescence normalized to vehicle-treated cells. Statistical significance was evaluated by one-way ANOVA followed by Bonferroni post hoc tests, indicating significant differences over vehicle-treated cells (***, *p* ≤ 0.001; ****, *p* ≤ 0.0001) and for peptide 10 over its related peptides (####, *p* ≤ 0.0001). Peptide 10 potency (*C*) was evaluated as in *A* over increasing peptide concentrations (*bottom*) (*n* = 3). Alternatively, to assess the kinetics of the peptide 10–driven heteromer disruption (*D*), prior to the administration of the peptide, the cells were preincubated with substrate, and the luminescence was recorded over the following 30 min. Data are mean RLU ± S.E. (*n* ≥ 3) percentage of luminescence normalized to vehicle-treated cells.

Next, we sought to test whether stapling changed the ability of the peptides to disrupt GPCR heteromers (*schematic* in Fig. 5*A*). Preincubation of HEK293 cells transiently co-expressing LgBiT–CB_1_R and SmBiT–5-HT_2A_R with the TM5-TAT and TM6-TAT, but not the TM7-TAT (negative control), peptides resulted in a decrease in the luminescence readout (Fig. 5*B*), corroborating the previously reported results demonstrating the involvement of TM5 and TM6, but not TM7, in the heteromeric interface (16). In addition, these results demonstrate the suitability of our NanoBiT-based peptide screening assay and the specificity of the detected interaction.

Next, we tested whether the stapled versions were as efficient at blocking heteromerization (Fig. 5*B*). When analyzing the TM5-TAT–derived peptides, compound **10** exerted a significant decrease in NanoBiT complementation. Interestingly, however, neither the version lacking the TAT amino acid sequence, compound **9**, nor the nonstaple, compound **11**, peptides induced any change. It was not simply length of the peptide, as the longer stapled compound **8** also was not as efficient as compound **10** at disrupting the complex. Stapling in itself did not seem to convey an advantage as, comparing compounds **9** and **11**, we observed equal effects.

We next sought to understand the disruption efficacy and the timing of compound **10**'s effects on the heteromer. Treatment with increasing concentrations of compound **10** induced a dose-dependent luminescence decrease, with a potency in the low micromolar range (pIC_50_ = 5.47 ± 0.01) (Fig. 5*C*). Surprisingly, the ability of compound **10** to disrupt 5-HT_2A_R–CB_1_R heteromers was on the order of minutes, reaching its maximal inhibitory response approximately 5 min after administration (Fig. 5*D*).

Altogether, by developing a sensitive and specific bimolecular luminescent complementation assay, we were able to screen a small library of peptides targeting 5-HT_2A_R–CB_1_R heteromers. In addition, covalent side chain–to–chain cross-linking through hydrocarbon peptide stapling led us to the identification of a small TM peptide mimetic, **10**, with improved stability, helicity, and efficacy.

## Discussion

Here we have successfully provided a proof of concept for two new tools to study GPCR oligomerization. First, we successfully applied the recently developed NanoBiT to study GPCR oligomerization. Using this system, we validated the previously demonstrated association of CB_1_R and 5-HT_2A_ receptors as homodimers and their ability to form heteromers (16, 31, 32). Second, we have demonstrated that interference peptides can be dramatically stabilized and shortened using aryl-carbon stapling. These two advances will prove useful in studying GPCR oligomerization both *in vitro* and particularly *in vivo*.

When adapting the NanoBiT to study oligomerization, we predicted that the small size of the complementary fragments (18 and 1.3 kDa for LgBiT and SmBiT, respectively), would minimize steric conflicts (17). In fact, agonist potencies and maximal efficacies were equivalent to their matched WT receptors under the same promoters. Apart from GLuc (19 kDa), NLuc (19 kDa) is significantly smaller compared with other fluorescent/luminescent proteins used in resonance energy transfer or protein complementation assays (ranging from 26 kDa for YFP up to 61 kDa for FLuc). In addition, no post-translational modifications have been reported in mammalian cells, resulting in lower energetic costs in terms of translation, sorting and proper polypeptide folding. Accordingly, our initial studies were performed under the control of the low-copy number herpes simplex virus thymidine kinase gene promoter (HSV-TK) (19). However, under this configuration, we were not able to quantitatively assess agonist-induced downstream signaling pathways. Importantly, most DNA constructs for FRET/BRET and BiFC assays use transient expression systems, such as pcDNA3.1 vectors, with the cDNA expressed under the control human CMV immediate-early enhancer and promoter (33, 34). Using this strategy, we developed a microtiter-based homogeneous assay that allowed the identification of GPCRs oligomers in just 24 h, reducing the chance of overexpression-related nonspecific interactions. When comparing our NanoBiT-based dimerization assay with Venus YFP BiFC, an approach used to visualize more than 200 PPIs, including many GPCR homo-/heteromers (22), NanoBiT BiLC proved far more sensitive at detecting CB_1_R and 5-HT_2A_R homodimers and 5-HT_2A_R–CB_1_R heteromers. Presumably, the small size of the fused fragments, NanoLuc high quantum yield, and the lack of BiFC maturation step act synergistically to allow us to detect PPIs 24 h after transfection under physiological conditions. More recently, TANGO and SPARK have been developed to study PPIs (35, 36). Both are good approaches for larger screens or, in the case of SPARK, enrichment of cell populations by FACS. The NanoBiT system adapted here is good for studies directly interrogating the interactions themselves, say for mapping the interactions themselves or testing known small molecules as we have shown in this study. Previously, a split NanoLuc system was published to study G-protein signaling, but it does not have the advantage of having a short peptide portion on one of the partners as is used in the NanoBiT system (37).

Interference peptides have been used in multiple studies to validate or disrupt GPCR heteromers (9, 13, 16, 38, 39). However, these peptides often suffer from poor pharmacokinetics and have a poor chance of being used or developed into druglike entities. To improve on the potential of such entities to be adapted as potential therapeutics, we have stabilized a disrupting peptide using hydrocarbon stapling. This approach has been used on a variety of peptide therapeutics. However, to our knowledge, stapled peptides have not been applied to G protein–coupled receptor oligomers. We demonstrate here that stapling of disrupting peptides can significantly shorten the required length of the peptide and dramatically improve the stability of the peptides. These data support further development of such an approach to target GPCR oligomers *in vivo*.

An additional challenge presented in targeting such complexes is that the same membrane polypeptide can often interact with multiple partners in different complexes (*e.g.* dopamine 2 receptor can interact with A2a receptor as well as dopamine 1 receptor, or different *N*-methyl-d-aspartate subunits or α-amino-3-hydroxy-5-methyl-4-isoxazolepropionic acid subunits can make up multiple complexes). To date, we have not seen that disrupting peptides are specific for a given complex. Our evidence here that additional modifications can be made to these peptides provides an opportunity for adding additional chemical modifications that might provide more complex specificity in the future.

## Experimental procedures

### Reagents

Unless stated otherwise, reagents and solvents were purchased as high-grade commercial products from Sigma-Aldrich. (*R*)-(+)-WIN 55212 and DOI were purchased from Tocris Bioscience.

### Expression vectors and cloning

Plasmids encoding the 3xHA-tagged human 5-HT_2A_ and CB_1_ receptors were obtained from the cDNA Resource Center (catalog numbers HTR02ATN01 and CNR010TN01, respectively). Plasmids encoding the pGloSensor^TM^-22F cAMP biosensor and the complementary NanoBiT pairs (pBiT1.1-C[TK/LgBiT], pBiT2.1-C [TK/SmBiT], pBiT1.1-N[TK/LgBiT], pBiT2.1-N[TK/SmBiT], LgBiT-PRKAR2A Control Vector, SmBiT-PRKACA Control Vector, and NanoBiT® Negative Control Vector) were purchased from Promega. The pGP-CMV-GCaMP6s calcium sensor was a gift from Douglas Kim and the GENIE Project (Addgene plasmid 40753). Plasmids encoding the Venus YFP complementary pairs pBiFC-bFosVC155 and pBiFC-bJunVN173 were a gift from Chang-Deng Hu (Addgene plasmids 22013 and 22012, respectively). All constructs generated in this study were generated following the Gibson assembly method according to the manufacturer's instructions (Gibson Assembly® Master Mix, New England Biolabs). Detailed information is provided in Table S1. The correct assembly of the full genes was verified by Sanger sequencing using universal T7 FW 5′-TAATACGACTCACTATAGGG-3′ and BGH RV 5′-TAGAAGGCACAGTCGAGG-3′ primers for constructs in pcDNA3.1(+) backbone, FW 5′-TTGGCAATCCGGTACTGTTGG-3′ and RV 5′-GCAATAGCATCACAAATTTC-3′ primers for constructs in pBiT1.1-C [TK/LgBiT] and pBiT1.1-N [TK/LgBiT] backbones, and FW 5′-TTGGCAATCCGGTACTGTGG-3′ and RV 5′-GCAATAGCATCACAAATTTC-3′ primers for constructs in pBiT2.1-C [TK/SmBiT] and pBiT2.1-N [TK/SmBiT] backbones.

### Cell culture and transfection

Human embryonic kidney 293 (HEK293) cells (ATCC® CRL-1573^TM^) were grown in Dulbecco's modified Eagle's medium supplemented with 2 mm l-glutamine, 4.5 g/liter d-glucose, 100 μg/ml sodium pyruvate, 100 units/ml penicillin, 100 μg/ml streptomycin, and 10% (v/v) heat-inactivated fetal bovine serum (PAN-Biotech, Aidenbach, Germany) at 37 °C in a 5% CO_2_ humidified atmosphere. Cells were directly transfected in a 96-well plate format following the reverse Lipofectamine^TM^ 3000 (Thermo Fisher Scientific) transfection method. Briefly, for each well, 100–200 ng of DNA and 0.2–0.4 μl of P3000^TM^ reagent (1:2 (w/v) DNA:P3000/Lipofectamine^TM^ 3000 ratio) were combined in 25 μl of Opti-MEM® medium. The total amount of DNA/well was kept constant with empty vector (pcDNA3.1). In a separate tube, 0.2–0.4 μl of Lipofectamine^TM^ was added to 25 μl of Opti-MEM® medium. Both reaction mixes were vortexed (2–5 s) and incubated at RT for 5 min. After this time, the diluted DNA was added dropwise to the Lipofectamine^TM^-containing tube, gently mixed by pipetting up and down, and incubated for 15 min at RT. In parallel, cells were trypsinized according to standard mammalian tissue culture protocols and resuspended in complete cell culture medium to 5 × 10^5^ viable cells/ml. 100 μl of the cell suspension was distributed into each well, and 50 μl of the transfection mix was added on top of the cells. The plates were then incubated at 37 °C in a 5% CO_2_ humidified atmosphere for 24–48 h before performing the experiments.

### Ca^2+^ and cAMP measurement

For [Ca^2+^]*_i_* release quantification, 50,000 cells/well were reverse-transfected (see above) with 50 ng/well of pGP-CMV-GCaMP6s calcium sensor vector and 100 ng/well of receptor in poly-d-lysine–coated black clear bottom 96-well plates. 24 h post-transfection, the cell culture medium was removed, and the cells were starved in FBS-free DMEM for 4 h at 37 °C in a 5% CO_2_ humidified atmosphere. Prior to [Ca^2+^]*_i_* release measurements, the cell culture medium was replaced by 175 μl of Ca^2+^ assay buffer (145 mm NaCl, 2.5 mm KCl, 10 mm glucose, 10 mm HEPES, 2 mm CaCl_2_, 1 mm MgCl_2_, pH 7.4), and the plates were pre-equilibrated for 1 h in the dark at 37 °C in a 5% CO_2_ humidified atmosphere. Immediately following agonist addition (25 μl, 8× final concentration), fluorescence emission intensity was recorded at 515 nm upon excitation at 488 nm in a CLARIOstar® multimode plate reader (BMG Labtech, Ortenberg, Germany) for 300 s every 5 s and 40 flashes/well at 37 °C. To account for differences in expression/cell density, an average of five prereadings were used to normalize each well's response.

cAMP measurements were performed as described previously (40). Briefly, 24 h post-transfection, cells were incubated in FBS-free DMEM for 4 h at 37 °C in a 5% CO_2_ humidified atmosphere. Prior to cAMP measurements, the cell culture medium was replaced, and plates were pre-equilibrated for 1 h with cAMP assay buffer (Hanks' balanced salt solution with 24 mm HEPES, 3.3 mm NaHCO_3_, 1.3 mm CaCl_2_, 1 mm MgSO_4_, 0.1% (w/v) BSA, pH 7.4) supplemented with 0.45 mg/ml firefly d-luciferin free acid. Immediately after agonist addition, luminescence was recorded using a CLARIOstar® multimode plate reader (BMG Labtech) with no lens (1-s integration time/well for 1 h every minute). To account for differences in expression/cell density, an average of six prereadings were used to normalize each well's response.

### NanoBiT BiLC and Venus BiFC assays

To assess GPCR protein–protein interactions with the NanoBiT technology, HEK293 cells (50,000 cells/well) were seeded in poly-d-lysine–coated white clear bottom 96-well plates and reverse-transfected (see above) with the plasmids encoding the complementary NanoBiT hemiprotein fragments. For orientation screenings, cells were transfected with two different concentrations (50 and 100 ng/well) of each receptor alone or in combination with the investigated partner (see figure legends) in the presence of empty vector (pcDNA3.1(+)) to normalize the total amount of DNA/well. For studies in the presence of increasing nontagged receptor competitors, 100 ng of each receptor pair (CB_1_R LgBiT + CB_1_R SmBiT, 5-HT_2A_R LgBiT + 5-HT_2A_R SmBiT, and 5-HT_2A_R LgBiT + CB_1_R SmBiT) were co-transfected with increasing concentrations of the different nontagged constructs (from 0 to 300 ng/well), and the total amounts of DNA/well were normalized with empty vector (pcDNA3.1(+)). For the screening of stapled peptides, 50 ng/well of both LgBiT CB_1_R and SmBiT 5-HT_2A_R were reverse-transfected as detailed previously. 24 h post- transfection, the cell culture medium was removed, and the cells were starved in FBS-free DMEM for 4 h at 37 °C in a 5% CO_2_ humidified atmosphere. The cell culture medium was replaced by 100 μl of NanoBiT assay buffer (same as for cAMP measurements), and the plates were pre-equilibrated for 1 h at RT in the dark. When peptide pretreatment was required, peptides were added over this pre-equilibration step, except when studying inhibition kinetics, when the peptides were administered immediately after the baseline luminescence recording. 25 μl/well of a 5× solution of the Nano-Glo® live cell reagent containing the cell-permeable furimazine substrate dissolved in Nano-Glo® LCS dilution buffer were added, and the luminescence was immediately monitored in CLARIOstar® Multimode Plate Reader (BMG Labtech) with no lens (1-s integration time/well for 1 h every minute).

For BiFC experiments, HEK293 cells growing at ∼80% confluence in 6-well plates were transfected with Lipofectamine^TM^ 3000 according to the manufacturer's instructions with 1.5 μg/well of both Venus YFP complementary plasmids and the corresponding individual receptor constructs. The total amount of DNA/well was kept constant with empty vector (pcDNA3.1(+)). 24 h post-transfection, the cells were trypsinized according to standard mammalian tissue culture protocols, and 5 × 10^5^ viable cells/ml were seeded in poly-d-lysine–coated black clear bottom 96-well plates and incubated at 37 °C in a 5% CO_2_ humidified atmosphere overnight. 48 h post- transfection, the cell culture medium was removed, and the cells were starved in FBS-free DMEM for 4 h at 37 °C in a 5% CO_2_ humidified atmosphere. Prior to fluorescence measurements, the cell culture medium was replaced with 100 μl of NanoBiT assay, and the plates were pre-equilibrated for 1 h at RT in the dark. Venus YFP fluorescence was measured at 530 nm (540–20 filter and 517.2-nm dichroic filter) upon excitation (40 flashes/well) at 489 nm (497–15 nm) in a CLARIOstar multimode plate reader (BMG Labtech) at 25 °C.

### Cell proliferation and toxicity assays

The iCELLigence real-time cell analyzer instrument (Roche Diagnostics GmbH and ACEA Biosciences) was used to analyze cell viability based on changes in electrical impedance over time (defined as cell index (CI)). Prior to the experiment, background CI levels of the 8-well E plate (ACEA Biosciences) were measured after the addition of 200 μl/well of prewarmed complete cell culture medium (see below). Immediately after, 200 μl of the cellular suspension (2.5 × 10^5^ viable HEK293 cells/ml) were distributed in each well, and cellular impedance was continuously monitored (time intervals are indicated in the respective figures) at 37 °C in a 5% CO_2_ humidified atmosphere. After 24 h, the E-plates were removed for peptide treatment (4 μm) and immediately returned back to the real-time cell analyzer, and CI changes were monitored under the same conditions over the next 48 h. Normalized cell index refers to the ratio between the CI values and CI from the time point immediately prior to ligand addition.

### Data analysis

Dose–response curves were fitted using a four-parameter logistic nonlinear regression mode. Peptide stability data were fitted using a nonlinear regression mode for dissociation kinetics. All statistical tests, curve fitting, and graphing were performed using GraphPad Prism 8 (GraphPad Software, La Jolla, CA). Information on the statistical test, significance, and experimental replicates is provided in the figure legends.

### Positioning of the staple position

We previously published a model of the CB_1_–5-HT_2A_ heterodimer (16). Using this model, we identified the amino acids of CB_1_R TM5 that would be facing outward from the CB1R receptor, and using an *i* + 3, we located the amino acids on which to place the staples using the logic that the staples should be on the opposite side of the helix from the interface of the two receptors.

### Synthesis of the unnatural amino acid

#### 

##### General procedure

All experiments were run under an atmosphere of nitrogen, using anhydrous solvents. All chemicals were purchased from Sigma-Aldrich. Analytical TLC was performed on Merck Kieselgel 60 F254 plates with visualization by UV light. Flash chromatography was performed on an Isolera^TM^ Prime (Biotage AB, Uppsala, Sweden). Melting points are uncorrected and were obtained in open capillaries using an electrothermal melting point apparatus. NMR spectra were recorded on Bruker DPX (^1^H, 400 MHz; ^13^C, 100 MHz; ^19^F, 376 MHz) spectrometers for CDC_l_3 solutions. NMR chemical shifts (δ) are given in ppm relative to CDCl_3_ at 7.26 ppm, and coupling constants (*J*) are reported in Hz. Spectral data are reported as follows: chemical shift, integration, multiplicity (s, singlet; d, doublet; t, triplet; m, multiplet). Optical rotations were measured on a JASCO P1010 polarimeter. High-resolution mass spectra were recorded at the National Mass Spectrometry Facility and Service at Swansea University Medical School (Swansea, UK).

##### Synthesis of (S)-1-(2-fluorobenzyl)pyrrolidine-2-carboxylic acid 1

l-Proline (8 g, 69.5 mmol) was added to a solution of freshly ground potassium hydroxide (11.7 g; 3 eq) previously dissolved in isopropyl alcohol (90 ml) at 40 °C. As soon as the solution became transparent, 2-fluorobenzyl bromide (8.5 ml) was added dropwise, and the solution was stirred for 18 h at 40 °C. Aqueous hydrochloric acid (37%) was added dropwise to the mixture until the solution reached pH 5–6, as determined using pH indicator strips. The suspension was then cooled in an ice bath for 15 min and filtered, and the precipitate was thoroughly washed with isopropyl alcohol. All filtrates were combined and concentrated *in vacuo* to give (*S*)-1-(2-fluorobenzyl)pyrrolidine-2-carboxylic acid **1** as a pale orange sticky compound (13.29 g, 93%). For analytical purposes, a small amount of BP was washed with acetone and concentrated *in vacuo* to afford a yellow solid powder of **1**. m.p: 79–81 °C; [α]D_20_ −23.9 (c 0.1 in MeOH); ^1^H NMR (400 MHz, CDCl_3_) δ 7.95 (1H, br s, OH), 7.42 (1H, d, *J* = 6.72 Hz, Ar-C*H*), 7.29–7.24 (1H, m, Ar-C*H*), 7.09 (1H, appt, *J* = 7.48 Hz, Ar-C*H*), 7.03 (1H, m, Ar-C*H*), 4.25 (1H, d, *J* = 13.04 Hz, N-C*H*_2_), 3.86 (1H, d, *J* = 13.05 Hz, N-C*H*_2_), 3.74 (1H, dd, *J* = 7.83, 6.3 Hz α-C*H*), 3.29 (1H, m, δ-C*H*_2_), 2.49 (1H, dd, *J* = 9.15, 17.85 Hz, δ-C*H*_2_), 2.21–2.01 (2H, m, β-C*H*_2_), 1.89–1.72 (2H, m, γ-C*H*_2_). ^13^C NMR (100 MHz; CDCl_3_) δ 175.41, 162.61, 132.76, 130.19, 124.36, 121.9, 115.9, 67.75, 52.83, 50.84, 29.12, 22.62. Additional peaks arise from rotamers at 130.11, 121.8, and 115.4; ^19^F NMR (376 MHz; CDCl_3_) −116.99; HRMS-ESI calculated for C_12_H_15_NO_2_F [M + H]^+^ 224.1087, found 224.1081.

##### Synthesis of (S)-N-(2-benzoylphenyl)-1-(2-fluorobenzyl)pyrrolidine-2-carboxamide 2

(*S*)-1-(2-Fluorobenzyl)pyrrolidine-2-carboxylic acid **1** (3.3 g, 14.73 mmol) was dissolved in CH_2_Cl_2_ (35 ml) at 0 °C. Methanesulfonyl chloride (5 ml, 14.73 mmol) and *N*-methylimidazole (2.6 ml, 32.4 ml) was added in a dropwise manner. After 5 min, 2-aminobenzophenone (2.62 g, 13.3 mol) was added, the ice bath was removed, and the reaction mixture was heated to 50 °C for 14 h. Saturated aqueous sodium hydrogen carbonate solution (30 ml) was added. The two layers were separated, and the aqueous layer was extracted with CH_2_Cl_2_ (3 × 30 ml). The organic extracts were combined, dried over sodium sulfate, filtered, and concentrated *in vacuo*. Purification by flash column chromatography (SiO_2_ eluted with 15% ethyl acetate-hexane) gave the title compound **2** as a pale yellow powder (2.94 g, 49.4%). m.p: 89–91 °C; [α]_D_^20^ −124.1 (c 0.25 in MeOH); ^1^H NMR (400 MHz, CDCl_3_) δ 11.43 (1H, s, NH), 8.56 (1H, dd, *J* = 8.19, 1.0 Hz, Ar-C*H*), 7.78–7.76 (2H, m, Ar-C*H*), 7.62 (1H, td, *J* = 7.41, 1.1 Hz), 7.55–7.48 (5H, m, Ar-C*H*), 7.11 (2H, m, Ar-C*H*), 6.94 (1H, td, *J* = 7.51, 1.2 Hz, Ar-C*H*), 6.80 (1H, dt, *J* = 9.22, 1.2 Hz, Ar-C*H*), 3.91 (1H, d, *J* = 13.52 Hz, N-C*H*_2_), 3.74 (1H, dd, *J* = 12.97, 1.2 Hz, N-C*H*_2_), 3.36 (1H, dd, *J* = 10.18, 4.46 Hz, α-C*H*), 3.24 (1H, m, β-C*H*_2_), 2.48 (1H, dd, *J* = 9.08, 16.54 Hz, β-C*H*2_)_, 2.26 (1H, m, δ-C*H*_2_), 1.96 (1H, d, *J* = 3.66, δ-C*H*_2_), 1.89–1.74 (2H, m, γ-C*H*_2_); ^13^C NMR (100 MHz; CDCl_3_) δ 198.1, 174.65, 160.02, 139.19, 138.8, 133.5, 132.7, 131.9, 130.3, 129.11, 129.03, 128.45, 125.8, 125.17, 125.02, 124.08, 122.5, 121.7, 115.42, 115.2, 68.11, 53.94, 52.2, 31.27, 24.45; ^19^F NMR (376 MHz; CDCl_3_) −117.6; HRMS-ESI (calculated for C_25_H_24_N_2_O_2_F [M + H]^+^ 403.1822, found 403.1816.

##### Synthesis of Ni-Ala-FBPB 3

(*S*)-*N*-(2-Benzoylphenyl)-1-(2-fluorobenzyl)pyrrolidine-2-carboxamide **2** (2.0 g, 4.96 mmol) was dissolved in methanol (55 ml) at 50 °C. Ni(NO_3_)_2_·6H_2_O (2.9 g, 9.93 mmol) and l-alanine (0.89 g, 9.93 mmol) were added to the reaction mixture, and after 3 min, freshly ground potassium hydroxide (1.95 g, 34.57 mmol) was added, and the mixture was heated to 70 °C for 1.5 h. The reaction mixture was cooled on the room temperature and concentrated. The residue was taken up in distilled water (50 ml) and extracted with EtOAc (3 × 50 ml). The combined organic layers were washed with brine solution (3 × 150 ml), dried over sodium sulfate, filtrated, concentrated *in vacuo*, and extensively washed with CHCl_3_ to give the title compound **3** as a red crystalline solid (2.61 g, 99%). m.p: 279–281 °C (lit^1^: 283–285 °C); [α]_D_^20^ +3432.9 (*c* 0.05 in CHCl_3_) (lit^1^: +3126.6 (*c* 0.05 in CHCl_3_); ^1^H NMR (400 MHz, CDCl_3_) δ 8.30 (1H, td, *J* = 7.52, 1.72 Hz, Ar-C*H*), 8.11 (1H, d, *J* = 8.52 Hz, Ar-C*H*), 7.53–7.49 (2H m, Ar-C*H*), 7.45 (1H, d, *J* = 7.46, Ar-C*H*), 7.24–7.20 (2H, m, Ar-C*H*), 7.19–7.12 (2H, m, Ar-C*H*), 7.05 (1H, appt, *J* = 9.49 Hz, Ar-C*H*), 6.95 (1H, d, *J* = 7.39, Ar-C*H*), 6.69–6.62 (2H, m, Ar-C*H*), 4.40 (1H, d, *J* = 13.06, N-C*H*H), 3.90 (1H, q, *J* = 7.01 Hz, α-C(Me)*H*), 3.82 (1H, d, *J* = 13.0Hz, N-CH*H*), 3.69 (1H, d, *J* = 6.71, β(Pro)-C*H*H), 3.51–3.46 (2H, m, α(Pro)-C*H*, γ(Pro)-CH*H*), 2.81 (1H, m, δ(Pro)-C*H*H), 2.56 (1H, m, δ(Pro)-CH*H*), 2.21 (1H, dt, *J* = 12.65, 6.50 Hz, γ(Pro)-CH*H*), 2.05 (1H, td, *J* = 11.59, 6.03 Hz, β(Pro)-CH*H*), 1.58 (3H, d, *J* = 7.03 Hz, C*H*_3_); ^13^C NMR (100 MHz, CDCl_3_) δ 180.49, 180.1, 170.35, 142.06, 134.21, 133.47, 133.2, 132.18, 131.29, 128.9, 127.5, 127.23, 126.62, 124.55, 123.92, 120.87, 120.87, 120.33, 120.30, 116.24, 116.02, 70.33, 66.64, 57.07, 55.6, 30.7, 24.16, 21.84; ^19^F NMR (376 MHz, CDCl_3_) δ −112.66; HRMS-ASAP calculated for C_28_H_27_N_3_O_3_FNi [M + H^+^] 532.1381, found 530.1357.

##### Synthesis of S5-Ni-Ala-FBFB 4

Finely ground sodium hydroxide (0.31 g, 7.52 mmol) was added to DMF (15 ml) under a nitrogen atmosphere with stirring at 5 °C. Ni-Ala-FBPB **3** (1 g, 1.88 mmol) was added, and the reaction mixture was stirred for 5 min. After the solution darkened in color, the ice bath was removed, and a solution of 1-bromo-4-pentene (0.873 g, 5.64 mmol) was added to the reaction mixture. The reaction mixture was heated to 50 °C and left to stir for 1 h. Upon completion of the reaction, the mixture was quenched with distilled water (10 ml). The mixture was concentrated *in vacuo*, taken up in distilled water (15 ml), and extracted with CH_2_Cl_2_ (3 × 20 ml). The combined organic extracts were washed with aqueous lithium chloride solution (5%, 3 × 40 ml) to thoroughly remove any DMF residue followed by brine (3 × 40 ml). The mixture was then dried over sodium sulfate and concentrated *in vacuo*. Purification by flash column chromatography (EtOAc-hexane = 1:1) gave the title compound **4** as a deep red-orange solid (0.69 g, 61%). m.p: 196–198 °C (lit^2^: 190–192 °C) [α]_D_^20^ +2201.1 (c 0.05, CHCl_3_) (lit^2^: +2271.2 (c 0.05, CHCl_3_); ^1^H NMR (400 MHz, CDCl_3_) δ 8.29 (1H, td, *J* = 7.4, 1.0 Hz, Ar-CH), 8.03 (1H, d, *J* = 8.5 Hz, Ar-C*H*), 7.51–7.44 (2H, m, Ar-C*H*), 7.38 (1H, m, Ar-C*H*), 7.33 (1H, m, Ar-C*H*), 7.29 (1H, m Ar-C*H*), 7.20 (1H, appt, *J* = 7.4 Hz, Ar-C*H*), 7.16 (1H, ddd, *J* = 8.4, 6.2, 2.2 Hz, Ar-C*H*), 7.06 (1H, appt, *J* = 9.1 Hz, Ar-C*H*), 6.97 (1H, d, *J* = 7.6 Hz, Ar-C*H*), 6.68–6.61 (2H, m, Ar-C*H*), 5.86 (1H, ddt, *J* = 17.0, 10.3, 6.5 Hz, C*H*CH_2_), 5.08 (1H, dd, *J* = 17.0, 1.0 Hz, CHC*H*_2_*cis*), 5.02 (1H, d, *J* = 10.3 Hz, CHC*H*_2_*trans*), 4.52 (1H, d, *J* = 13.1 Hz, N-C*H*H), 3.95 (1H, d, *J* = 13.1 Hz, N-CH*H*), 3.60 (1H, dd, *J* = 9.9, 6.5 Hz, α(Pro)-C*H*), 3.41 (1H, dd, *J* = 10.7, 6.4 Hz, δ(Pro)-C*H*H), 3.26 (1H, m, β(Pro)-C*H*H), 2.78 (1H, m, γ(Pro)-C*H*H), 2.52 (1H, m, γ(Pro)-CH*H*), 2.40 (1H, m, γ-C*H*H), 2.17–1.98 (5H, m, δ-C*H*2, γ-CH*H*, δ(Pro)-CH*H*, β(Pro)-CH*H*), 1.75–1.62 (2H, m, β-C*H*_2_), 1.23 (3H, s, C*H*_3_); ^13^C NMR (101 MHz, CDCl_3_) δ 182.38, 180.15, 172.4, 141.5, 137.83, 136.44, 134.2, 133.45, 131.62, 131.31, 130.32, 129.41, 128.77, 127.97, 127.34, 126.94, 124.51, 124.0, 120.8, 120.33, 116.28, 116.0, 115.44, 78.1, 70.15, 56.67, 55.86, 39.78, 33.71, 30.54, 29.6, 25.27, 23.25; ^19^F NMR (376 MHz, CDCl_3_) δ −113.7; HRMS-ESI calculated for C_33_H_35_N_3_O_3_FNi [M + H]^+^ 598.2016, found 598.6649.

##### Synthesis of S-pentenylalanine 5

3 m hydrochloric acid (3.1 ml, 24 eq) was warmed to 70 °C. A solution of S5-Ni-Ala-FBFB **4** (300 mg) dissolved in methanol (25 ml) was added dropwise to prewarmed HCl. In 5 min, a color change from red to transparent green/yellow was observed. The mixture was left stirring for an additional 20 min and then cooled to room temperature. After removing the methanol *in vacuo*, the residue was taken up in water (20 ml) and extracted with DCM (3 × 20 ml). The organic extracts were combined, dried (MgSO_4_), and concentrated *in vacuo* to reclaim **2**. A few drops of 1 m NaOH were added to the green aqueous solution to precipitate nickel-salts. After 2 min of centrifugation at 600 rpm, the filtrate was decanted, and after removal of the water *in vacuo*, *S*-pentenylalanine **5** was isolated as a white powder (70 mg, 89%). m.p: 242–244 °C (lit^2^: 250–252 °C) [α]_D_^20^ +3.09 (c 0.05, MeOH) (lit^2^: +3.22 (c 0.05, MeOH, 25 °C)); ^1^H NMR (400 MHz, D_2_O) δ 5.74 (1 H, dd, *J* = 10.23, 17.08 Hz, CHCH_2_), 4.94 (2 H, m, CHCH_2_), 1.99 (2 H, d, *J* = 6.55 Hz, δ-CH_2_), 1.85 (1 H, m, β-CHH), 1.74 (1 H, m, β-CHH), 1.50–1.35 (4 H, m, CH_3_, γ-CHH), 1.26 (1 H, m, γ-CHH); ^13^C NMR (100 MHz, D_2_O) δ 177.0, 138.7, 115, 61.6, 36.7, 32.8, 22.5, 22.5; HRMS-ESI (calculated for C_8_H_16_NO_2_ [M + H^+^] 158.1181, found 158.1562.

##### Fmoc–S-pentenylalanine 6

Potassium carbonate (70 mg, 0.51 mmol) and *S*-pentenylalanine **5** (40 mg, 0.25 mmol) was dissolved in water (1 ml) and cooled to 0 °C. pH was routinely checked with the pH indicators, and after confirming the basic conditions, Fmoc-Cl (100 mg, 0.38 mmol) was dissolved in dioxane (1.4 ml) and added to the reaction mixture over 10 min. The reaction was then warmed to room temperature and stirred for 36 h. An excess volume of water was added, and the mixture was extracted with ethyl acetate (2 × 10 ml). The combined organic phases were then extracted with saturated bicarbonate solution (2 × 20 ml), and the aqueous layer acidified to pH 1 with 6 m HCl. The combined aqueous phases were then extracted with EtOAc (3 × 20 ml). The organic phases were combined, dried (MgSO_4_), and concentrated *in vacuo*. Purification by column chromatography (SiO_2_ eluted with MeOH/CH_2_Cl_2_/AcOH (97:2:1) gave Fmoc–*S*-pentenylalanine **6** as a white powder (35 mg, 21%). m.p: 225–227 °C [α]^D^_20_ +3.6 (c 1.0, MeOH) (lit^2^: +3.5 (c 1.0, MeOH, 25 °C); ^1^H NMR (400 MHz, CDCl_3_) δ 7.77 (dd, *J* = 7.3, 3.7 Hz, 2H), 7.59 (s, 2H), 7.45–7.38 (m, 2H), 7.35–7.28 (m, 2H), 5.75 (s, 1H), 5.46 (s, 1H), 4.99 (t, *J* = 14.2 Hz, 2H), 4.42 (s, 2H), 4.22 (s, 1H), 2.05 (s, 2H), 1.86 (s, 1H), 1.60 (s, 3H), 1.27 (s, 2H). ^13^C NMR (101 MHz, CDCl_3_) δ 179.18, 154.82, 143.81, 141.37, 138.02, 127.72, 127.09, 125.02, 120.01, 115.10, 66.56, 59.76, 47.23, 36.22, 33.39, 23.29; HRMS-ESI (calculated for C_23_H_26_NO_4_ [M + H^+^] 380.1862, found 380.1860.

### Synthesis and purification of the TM peptides

TM peptides were synthesized on a fully automated Syro I (Biotage) instrument using standard procedures for Fmoc-solid phase peptide synthesis on a 100-mg preloaded H-Arg(Pbf)-HMPB NovaPEG or H-Phe-HMPB NovaPEG resin (Merck Millipore, Darmstadt, Germany) with resin loading of 0.48 or 0.63 mmol/g, respectively, as a solid support. The preloaded arginine resin was swollen in DMF for 20 min and checked for potential clumping because clumped resin may impact the synthesis. After the swelling, coupling was achieved using 4 eq of the following: Fmoc-protected amino acid, HOBt (hydroxybenzotriazole) and HBTU (2-(1H-benzotriazol-1-yl)-1,1,3,3-tetramethyluronium), and 8 eq of DIPEA (*N*,*N*-diisopropylethylamine) in NMP. Each coupling reaction was left for 45 min and performed twice to ensure complete coupling. Fmoc deprotection was achieved using 40% piperidine in DMF (3 × 1.5 ml). After final incorporation of the last amino acid, the Fmoc group was removed, and the resin was washed thoroughly with DMF, DCM, and 1:1 DCM/MeOH to remove any residual DMF and dried *in vacuo*. Cleavage from the solid support was carried out using TFA/water/ethanedithiol/TIPS (v/v/v/v = 94:2.5:2.5:1, 10 ml/0.1 g resin) in the case of TM5–7 for 3 h. For peptides **7–11**, TFA/water/TIPS (v/v/v = 95/2.5/2.5, 10 ml/0.1 g resin) was used. The resin was washed with TFA (10 ml), combined with the cleavage mixture, and concentrated *in vacuo*. The crude peptide was precipitated with a 10-fold excess of cooled (−20 °C) diethyl ether, leaving a white precipitate.

Following crude peptide isolation, the peptides were dissolved in methanol (40 ml), filtered, and purified by preparative RP-HPLC (Agilent 1200 Infinity) using an Aeris PEPTIDE 5-μm XB-C18 column, 150 × 21.2 mm with a gradient of 95:5 water/MeOH with 0.05% TFA to 5:95 water/MeOH over 15 min returning to 95:5 water/MeOH over 5 min at a flow rate of 20 ml/min at the detection wavelength of 214 nm. Analytical RP-HPLC was then used to assess the purity of the products after the purification and was performed on an Agilent 1200 using an Aeris Peptide 5-μm XB-C18 column, 150 × 4.6 mm, with the same conditions as above. Following purification, the peptides were concentrated, resuspended in distilled water, and lyophilized. Mass analysis was performed to determine the identities of TM peptides by MALDI-TOF MS, using sinapinic acid as the ionization matrix.

### Synthesis and purification of the stapled peptides

Peptides were synthesized on a Syro I fully automated peptide synthesizer using Rink amide LL 100–200 mesh resin (0.36 mmol/g) at 36-μmol scale as described above. Coupling frequency and incubation times were 2 × 45 min for standard amino acid residues, 2 × 60 min for the olefinic nonnatural amino acid **6**, and 2 × 60 min for the residue following a nonnatural amino acid. After the automated synthesis, the ring-closing metathesis was performed on the peptide while still on the solid support in a disposable fritted reaction vessel. The peptide resin was cyclized in the presence of Grubbs catalyst second-generation catalyst (10 mm) in anhydrous 1,2-dichloroethane (DCE) for 4 h at room temperature. Completeness of the RCM reaction was monitored by HPLC. The resin- bound peptide was washed with DCE and then with DCM, DCM/MeOH (1:1) and dried under vacuum. The Fmoc group was removed with 30% piperidine in DMF (2 × 10 min), washed with DMF, DCM, DCM/MeOH (1:1), and dried under vacuum. The stapled peptides were cleaved from the solid support by treatment with TFA/H_2_O/TIPS (95:2.5:2.5) for 2–3 h at room temperature. The resin was washed with TFA (10 ml), combined with the cleavage mixture, and concentrated *in vacuo*. The crude peptide was precipitated with a 10-fold excess of cooled (−20 °C) diethyl ether, leaving a white precipitate. Purification, HPLC, and MALDI-TOF analysis were performed as above.

### Monitoring the RCM reaction

An analytical test for monitoring the progress of the RCM reaction was performed in a similar manner as described by Young Woo *et al.* (24). A 50-μl aliquot of resin suspension was taken out of the reaction and washed with 200 μl of anhydrous DCE under nitrogen bubbling. After that initial wash, the reaction solution was washed with DCM and DCM/MeOH (1:1), cleaved with 60 μl of cleavage mixture (TFA/H20/TIS (95:2.5:2.5)) for 40 min, resin was removed, TFA was evaporated, and the peptide was precipitated with cold diethyl ether. The pellet was left to air-dry before dissolving it in 25 μl of 95:5 water/MeOH with 0.05% TFA and analyzed using analytical RP-HPLC.

### CD helicity measurements

CD spectra were recorded on a Jasco J-810 with a total of three scans from 195 to 260 nm in 0.5-nm increments. The averaged scans were collected at a scanning speed of 200 nm/min using a 1-mm path length cell. Peptides were prepared as described in Greenfield's protocol (41) and dissolved in MilliQ deionized water with the target concentration between 30 and 50 μm with the exact concentration then confirmed using a BCA protein assay kit according to the manufacturer's protocol. The CD spectrum of the MilliQ Water was subtracted from the spectrum of the sample.

The Jasco J-810 generates the raw output in ellipticity and is measured in millidegrees. This was first converted to molar ellipticity ([θ]) with units of (*d* × *c*^2^)/*d*. Once the precise concentration was confirmed, the molar ellipticity [θ] was calculated as follows.

(Eq. 1) $$\left[\theta \right]=\frac{\theta_{o}\times 10^{6}}{c\times l\times n}$$

Where *c* is the sample concentration (μm), *l* is the path length (mm), *n* is the number of peptide bonds (calculated as amino acid residues − 1), and θ_obs_ is the observed ellipticity (millidegrees). To calculate the percentage of helicity, the mean residue ellipticity and θ_MAX_ were calculated according to Forood *et al.* (42) and others (43–45), where θ_222_ is molar ellipticity at 222 nm, *c* is molar concentration of the peptide, and *n* is the number of amino acid residues,

(Eq. 2) $${\left[\theta \right]}_{m}=\left(-44,000+250T\right)\times \left(1-\frac{k}{n}\right)$$

where *T* is the temperature (273 K) and *k* is the number of nonhydrogen-bonded peptide carbonyls. According to Shepherd *et al.* (44), intramolecular hydrogen bonds are characteristic of α helicity, so inclusion/exclusion of an N-terminal acetyl group or C-terminal amide group affects *k*. For Ac-[peptide]-NH_2_, *k* = 3; for H-[peptide]-NH_2_, *k* = 2; and for H-[peptide]-OH *k* = 1.

Percentage helicity was then calculated as follows.

(Eq. 3) $$\%\;\text{helicity}=\frac{{\left[\theta \right]}_{2}}{{\left[\theta \right]}_{m}}\times 100$$

### Proteolytic stability studies

#### 

##### Stability against trypsin

To 100 μl of peptide solution (100 μm, dissolved in ammonium bicarbonate buffer, pH 7.5), 60 μl of ammonium bicarbonate buffer (pH 7.5) was added, together with 20 μl of temperature-equilibrated (37 ± 1 °C) trypsin (5 μg/ml) from porcine pancreas (Sigma, 13,000–20,000 BAEE units/mg of protein). Peptides were incubated for 15, 30, 60, 120, 240, and 480 min, and then MeOH (HPLC Grade) + 0.05% TFA was added. The samples were then centrifuged (15,000 rpm), and the supernatant was analyzed, using Fmoc-Gly (10 μl, 0.2 mm) as an internal standard. The digestion at each time point was repeated three times to give the average values along with S.D. The amount of intact peptide that remained in the mixture was quantified by RP-HPLC. The experiment was repeated twice on different days.

##### Stability against chymotrypsin

To 100 μl of peptide solution (100 μm, dissolved in ammonium bicarbonate buffer, pH 7.5), 60 μl of ammonium bicarbonate buffer (pH 7.5) was added, together with 20 μl of temperature-equilibrated (37 ± 1 °C) α-chymotrypsin (5 μg/ml) from bovine pancreas (Sigma, Type II, activity > 40 units/mg protein). Peptides were incubated for 15, 30, 60, 120, 240, and 480 min, and then MeOH (HPLC grade) + 0.05% TFA was added. The samples were then centrifuged (15,000 rpm), and supernatant was analyzed in a similar manner as described above. The experiment was repeated twice on different days.

##### Stability in the mouse serum

To 200 μl of fresh nonsterile mouse serum was added 25 μl of peptide solution (100 μm, dissolved in ammonium bicarbonate buffer, pH 7.5, containing 10% DMSO), and the mixture was incubated at 37 °C. At the specified time, an aliquot of incubation mixture was withdrawn and quenched by the addition of an equal volume of 15% TCA in acetonitrile to precipitate out serum proteins over ice for 30 min. The mixture was then centrifuged at 13,500 rpm for 10 min, and the supernatant was collected and analyzed by HPLC in a similar manner as described previously. The experiment was repeated twice on different days.

## Author contributions

J. B. and P. J. M. data curation; J. B., P. J. M., and L. A. H. formal analysis; J. B. validation; J. B., L. B., and P. K. investigation; J. B., L. B., P. K., and L. A. H. methodology; J. B., P. J. M., and L. A. H. writing-original draft; J. B., P. J. M., and L. A. H. writing-review and editing; P. K. resources; P. J. M. and L. A. H. conceptualization; P. J. M. and L. A. H. supervision; P. J. M. and L. A. H. funding acquisition; P. J. M. project administration.

## Supplementary Material

Supporting Information

## Footnotes

4 J. Botta, L. Bibic, P. Killoran, P. J. McCormick, and L. A. Howell, unpublished observations.

## Footnotes

3 The abbreviations used are:

GPCR: G-protein–coupled receptor
TM: transmembrane domain
CB_1_R: cannabinoid receptor 1
5-HT_2A_: serotonin receptor type 2A
TIPS: triisopropylsilane
AT1aR: angiotensin receptor subtype 1a
THC: *trans*-Δ9-tetrahydrocannabinol
NanoBiT: NanoLuc binary technology
CMV: cytomegalovirus
PPI: protein–protein interaction
RFU: relative fluorescence units
RLU: relative luminescence units
DOI: (±)-2,5-dimethoxy-4-iodoamphetamine hydrochloride
Fmoc: *N*-(9-fluorenyl)methoxycarbonyl
S_5_-OH: *S*-pentenylalanine
RCM: ring-closing metathesis
RP: reversed-phase
RT: room temperature
DMEM: Dulbecco's modified Eagle's medium
CI: cell index
DMF: *N*,*N*-dimethylformamide
DCM: dichloromethane
HRMS: high-resolution MS
ESI: electrospray ionization
TFA: trifluoroacetic acid
DCE: 1,2-dichloroethane
ANOVA: analysis of variance
BiFC: bimolecular fluorescent complementation
YFP: yellow fluorescent protein
BiLC: bimolecular luminescent complementation.
