# Supplementary material for: Design and development of stapled transmembrane peptides that disrupt the activity of G-protein–coupled receptor oligomers
Source: J Biol Chem. 2019 Aug 29;294(45):16587–603. doi: 10.1074/jbc.RA119.009160 (PMC6851324; doi:10.1074/jbc.RA119.009160)
Supplement: Supporting Information [file supp_294_45_16587__index.html]

Design and development of stapled transmembrane peptides that disrupt the activity of G-protein coupled receptor oligomers — Stapled TM peptides modulate GPCR oligomers — Design and development of stapled transmembrane peptides that disrupt the activity of G-protein–coupled receptor oligomers — Stapled TM peptides modulate GPCR oligomers — Supporting Information 

# Design and development of stapled transmembrane peptides that disrupt the activity of G-protein–coupled receptor oligomers

## Supporting Information

- Supporting Information (to be published online) - Supporting figures and materials and methods
