# Supplementary material for: Design and development of stapled transmembrane peptides that disrupt the activity of G-protein–coupled receptor oligomers
Source: J Biol Chem. 2019 Aug 29;294(45):16587–603. doi: 10.1074/jbc.RA119.009160 (PMC6851324; doi:10.1074/jbc.RA119.009160)
Supplement: Supporting Information [file supp_RA119.009160_152553_1_supp_385294_pwy89y.pdf]

Supplementary Information

Design and development of stapled transmembrane peptides that disrupt the activity of G-protein coupled receptor oligomers

Joaquín Botta[1,2], Lucka Bibic[2], Patrick Killoran[3], Peter J. McCormick*[1] and Lesley A. Howell*[4]

From the [1]Centre for Endocrinology, William Harvey Research Institute, Bart's and The London School of Medicine and Dentistry, Queen Mary University of London, Charterhouse Square, London, EC1M 6BQ; [2]School of Pharmacy, University of East Anglia, Norwich Research Park, Norwich, NR4 7TJ; [3]School of Pharmacy and Biomolecular Sciences, James Parsons Building, Byrom Street, Liverpool, L3 3AF; [4]School of Biological and Chemical Sciences, Queen Mary University of London, Mile End Road, London, E1 4NS

Running Title: *Stapled TM peptides modulate GPCR oligomers*

* To whom correspondence should be addressed: Lesley A. Howell: School of Biological and Chemical Sciences, Queen Mary University of London, Mile End Road, London, E1 4NS; l.howell@qmul.ac.uk; Tel. (+44) 207 8826625 or Peter J. McCormick: Centre for Endocrinology, William Harvey Research Institute, Bart's and The London School of Medicine and Dentistry, Queen Mary University of London, Charterhouse Square, London, EC1M 6BQ; p.mccormick@qmul.ac.uk.

Keywords: G protein-coupled receptor (GPCR), cannabinoid receptor type 1 (CB_1), dimerization cell-penetrating peptide (CPP), peptide chemical synthesis, serotonin receptor type 2A ($5HT_{2A}$), NanoLuc binary technology (NanoBiT), hydrocarbon stapling, transmembrane peptide, bimolecular fluorescence complementation

---

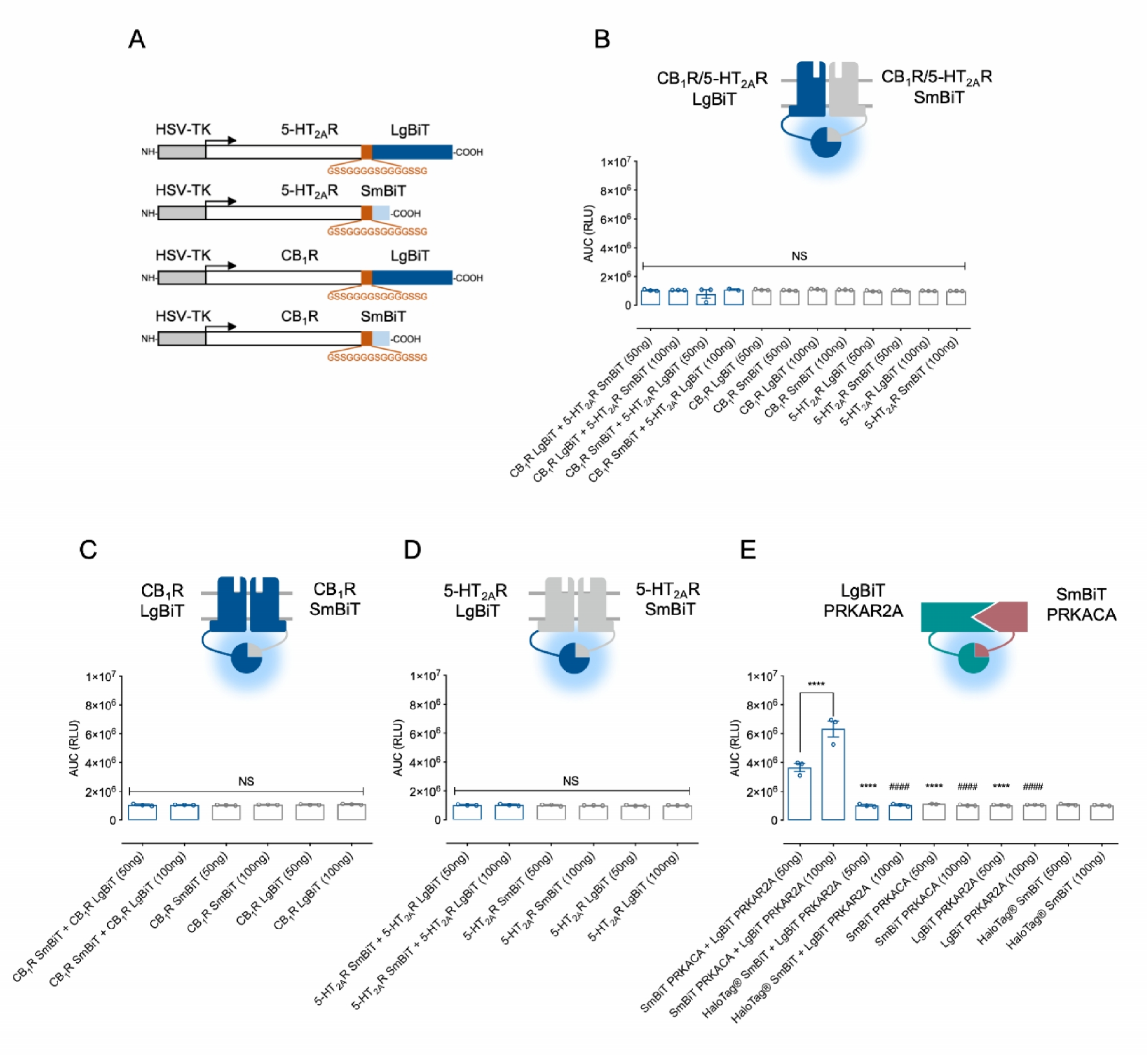

Supplementary figure 1. Conformational screening of the optimal orientation for CB$_1$, 5-HT$_{2A}$ and 5-HT$_{2A}$-CB$_1$ receptor oligomers. HSV-TK promoter studies. (A) Schematic representation of the NanoLuc fusion proteins. HEK-293 cells were transiently transfected with all possible orientations of LgBiT and SmBiT C-terminal fusions at two different DNA ratios (50 or 100 ng receptor/well) to assess 5-HT$_{2A}$R-CB$_1$R heteromers **(B)** or CB$_1$R **(C)** and 5-HT$_{2A}$R **(D)** homomers. In **(E)**, the SmBiT-PRKACA and LgBiT-PRKAR2A positive interacting control was transfected under the same conditions. The LgBiT-PRKAR2A plus HaloTag®-SmBiT combination was used as a non-interacting negative control. Grey bars represent the RLU of each individual construct expressed alone. Data are mean AUC (RLU) ± SEM (n = 3). Statistical significance was evaluated by one-way analysis of variance (ANOVA) followed by Bonferroni post hoc tests showing significant effects over SmBiT PRKACA + LgBiT PRKAR2A (50 ng) (****p ≤0.0001) or over SmBiT PRKACA + LgBiT PRKAR2A (100 ng) (####p ≤0.0001). HSV-TK, herpes simplex virus thymidine kinase gene promoter; NS, non-statistical significant.

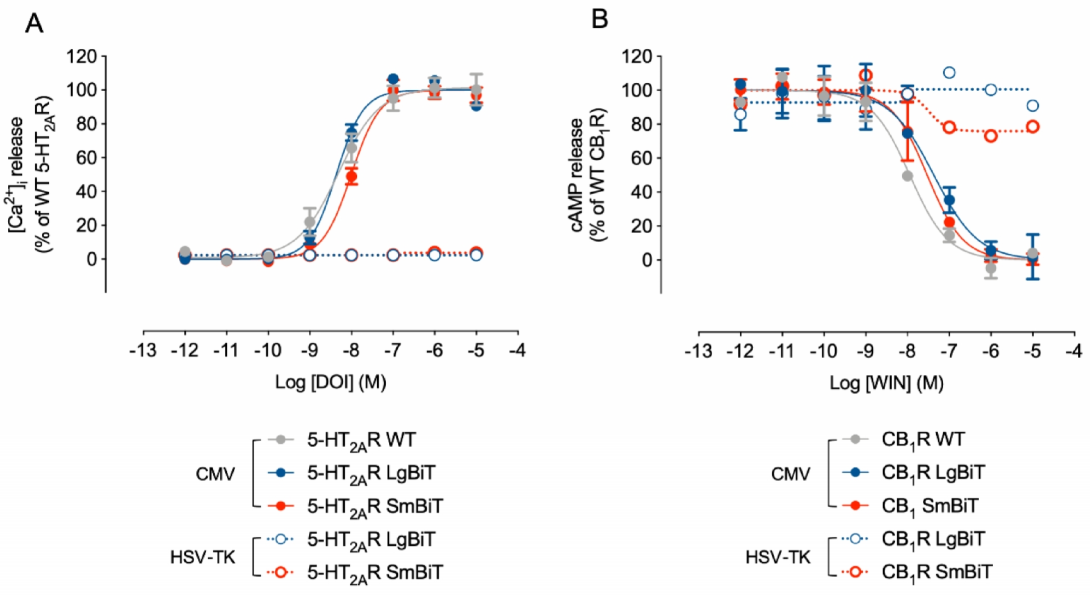

	CMV promoter	HSV-TK promoter
**5-HT$_{2A}$R WT**	8.31 ± 0.13	-
**5-HT$_{2A}$R LgBiT**	8.37 ± 0.06	N.D.
**5-HT$_{2A}$R SmBiT**	8.01 ± 0.06	N.D.
**CB$_1$R WT**	7.95 ± 0.14	-
**CB$_1$R LgBiT**	7.37 ± 0.16	N.D.
**CB$_1$R SmBiT**	7.52 ± 0.09	7.51 ± 0.35

Supplementary figure 2. Functional validation of NanoBiT C-terminal fusions. In **(A)**, dose-response intracellular Ca^{2+} release curves for 5-HT$_{2A}$R constructs. In **(B)**, forskolin-induced (7.5µM) cAMP release inhibition dose-response curves for CB$_1$R. Data are mean ± SD (n≥2) percentage of activation normalised to each wild type receptor maximal response. The bottom table represents the mean pEC$_{50}$/pIC50 ± SD (n≥2). HSV-TK, herpes simplex virus thymidine kinase gene promoter; CMV, human cytomegalovirus immediate-early promoter. ND, non-determined, indicates non-convergent curve fits.

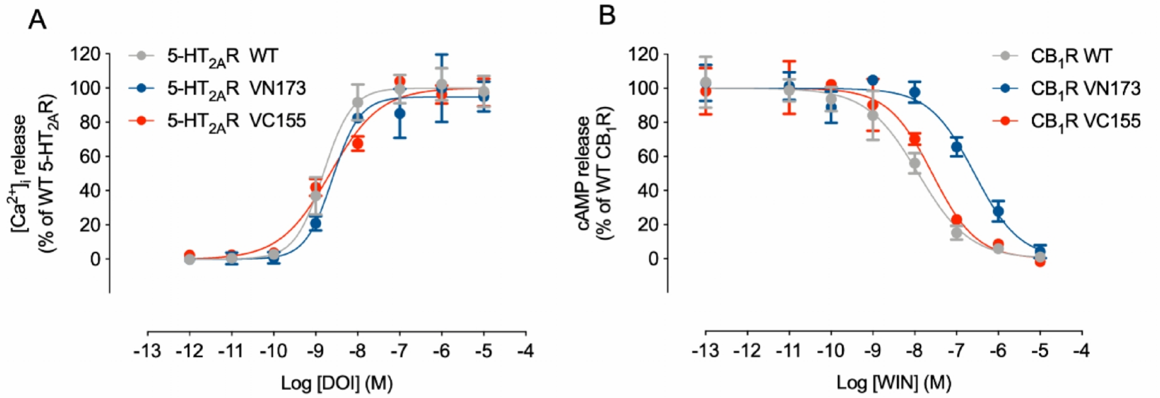

	pEC/IC_{50}
**5-HT$_{2A}$R WT**	8.82 ± 0.11
**5-HT$_{2A}$R VN173**	8.60 ± 0.16
**5-HT$_{2A}$R VC155**	8.66 ± 0.13
**CB$_1$R WT**	7.92 ± 0.13
**CB$_1$R VN173**	6.57 ± 0.16**
**CB$_1$R VC155**	7.58 ± 0.20

Supplementary figure 3. Functional validation of split Venus YFP C-terminal fusions. In **(A)**, dose-response intracellular Ca^{2+} release curves for 5-HT$_{2A}$R constructs. In **(B)**, forskolin-induced (7.5μM) cAMP release inhibition dose-response curves for CB$_1$R. Data are mean ± SD (n≥2) percentage of activation normalised to each wild type receptor maximal response. The bottom table represents the mean pEC$_{50}$/pIC50 ± SD (n≥2). Statistical significance was evaluated by one-way ANOVA followed by Bonferroni post hoc tests indicating significant differences over its respective WT receptor (**p ≤0.01).

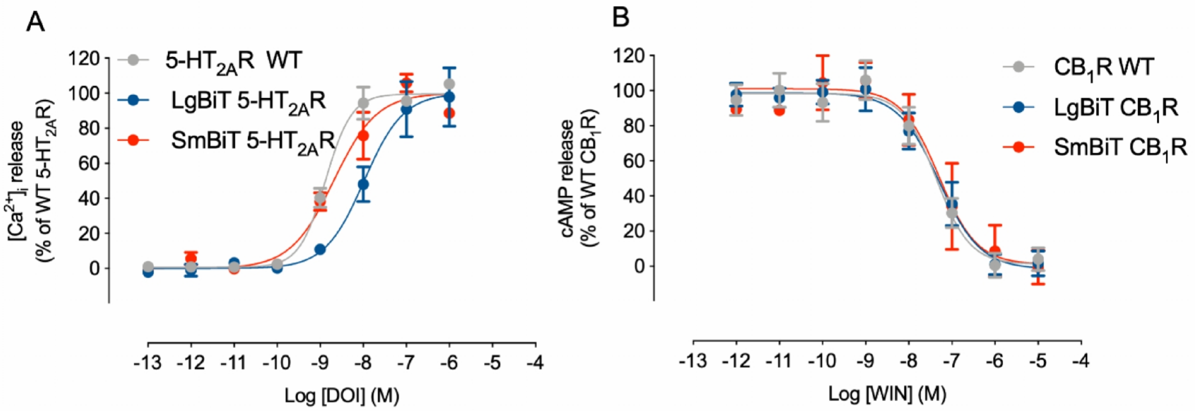

	pEC/IC$_{50}$
**5-HT$_{2A}$R WT**	8.88 ± 0.09
**LgBiT 5-HT$_{2A}$R**	7.98 ± 0.16*
**SmBiT 5-HT$_{2A}$R**	8.69 ± 0.16
**CB$_1$R WT**	7.36 ± 0.18
**LgBiT CB$_1$R**	7.30 ± 0.15
**SmBiT CB$_1$R**	7.30 ± 0.17

Supplementary figure 4. Functional validation of NanoBiT N-terminal fusions. In **(A)**, dose-response intracellular Ca^{2+} release curves for 5-HT$_{2A}$R constructs. In **(B)**, forskolin-induced (7.5μM) cAMP release inhibition dose-response curves for CB$_1$R. Data are mean ± SD (n≥2) percentage of activation normalised to each wild type receptor maximal response. The bottom table represents the mean pEC$_{50}$/pIC50 ± SD (n≥2). Statistical significance was evaluated by one-way ANOVA followed by Bonferroni post hoc tests indicating significant differences over its respective WT receptor (*p ≤0.05).

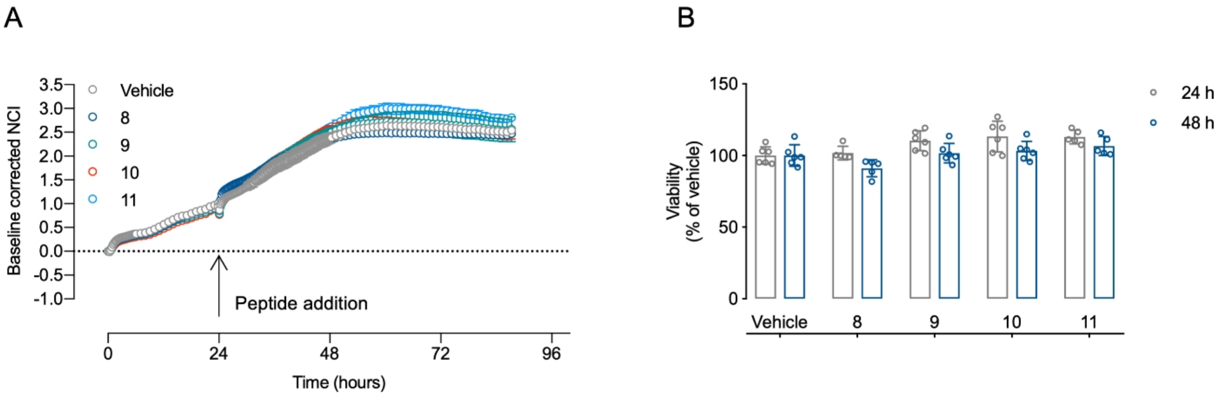

Supplementary figure 5. Label-free real-time evaluation of peptide toxicity. (A) Representative real-time iCELLigence RTCA impedance traces monitoring cell growth after peptide (4 μM) addition. Data are shown as the mean baseline corrected NCI ± SD (n=2-3). In **(B)**, bar graphs plotting cell index values 24- and 48-hours post-peptide addition. Data are mean ± SEM (n≥5) normalised to vehicle-treated cells. Statistical significance was evaluated by two-way analysis of variance (ANOVA) followed by Bonferroni post hoc tests showing no statistically significant reduction in viability.

Supplementary table 1.

Plasmid name	Backbone	Backbone primers (5’ 3’)		Insert	Insert primers (5’ 3’)	
**HSV-TK-3xHA-5-HT$_{2A}$R-LgBiT**	pBiT1.1-C [TK/LgBiT]	FW	CTGTGTGGGCTCGAGCG GTGGTGGC	pcDNA3.1(+)-3xHA-5-HT$_{2A}$R	FW	GGAATTCTGGTACCACCATGT ACCCATACGA
		RV	GGTGGTACCAGAATTCC CCTGAGCTCC		RV	CTCGAGCCCACACAGCTCACC TTTTCATTCACTCC
MYPYDVPDYAYPYDVPDYAYPYDVPDYADDILCEENTSLSSTTNSLMQLNDDTRLYSNDFNSGEANTSDAFNWTVDSENRTNLSCE<br>GCLSPSCLSLLHLQEKNWSALLTAVVIILTIAGNILVIMAVSLEKKLQNATNYFLMSLAIADMLLGFLVMPVSMLTILYGYRWPLPSKLCA<br>VWIYLDVLFSTASIMHLCAISLDRYVAIQNPIHHSRFNSRTKAFLKIIAVWTISVGISMPIPVFGLQDDSKVFKEGSCLLADDNFVLIGSFV<br>SFFIPLTIMVITYFLTIKSLQKEATLCVSDLGTRAKLASFSFLPQSSLSSEKLFQRSIHREPGSYTGRRTMQSISNEQKACKVLGIVFFLFV<br>VMWCPFFITNIMAVICKESCNEDVIGALLNVFVWIGYLSSAVNPLVYTLFNKTYRSAFSRYIQCQYKENKKPLQLILVNTIPALAYKSSQL<br>QMGQKKNSKQDAKTTDNDCSMVALGKQHSEEASKDNSDGVNEKVSCVGSSGGGGSGGGGSSGVFTLEDFVGDWEQTAAYNLDQ<br>VLEQGGVSSLLQNLAVSVTPIQRIVRSGENALKIDIHVIIPYEGLSADQMAQIEEVFKVVYPVDDHHFKVILPYGTLVIDGVTPNMLNYFG<br>RPYEGIAVFDGKKITVTGTLWNGNKIIDERLITPDGSMLFRVTINS						
**HSV-TK-3xHA-5-HT$_{2A}$R-SmBiT**	pBiT2.1-C [TK/SmBiT]	FW	CTGTGTGGGCTCGAGCG GTGGTGGC	pcDNA3.1(+)-3xHA-5-HT$_{2A}$R	FW	GGAATTCTGGTACCACCATGT ACCCATACGA
		RV	GGTGGTACCAGAATTCC CCTGAGCTCC		RV	CTCGAGCCCACACAGCTCACC TTTTCATTCACTCC
MYPYDVPDYAYPYDVPDYAYPYDVPDYADDILCEENTSLSSTTNSLMQLNDDTRLYSNDFNSGEANTSDAFNWTVDSENRTNLSCE<br>GCLSPSCLSLLHLQEKNWSALLTAVVIILTIAGNILVIMAVSLEKKLQNATNYFLMSLAIADMLLGFLVMPVSMLTILYGYRWPLPSKLCA<br>VWIYLDVLFSTASIMHLCAISLDRYVAIQNPIHHSRFNSRTKAFLKIIAVWTISVGISMPIPVFGLQDDSKVFKEGSCLLADDNFVLIGSFV<br>SFFIPLTIMVITYFLTIKSLQKEATLCVSDLGTRAKLASFSFLPQSSLSSEKLFQRSIHREPGSYTGRRTMQSISNEQKACKVLGIVFFLFV<br>VMWCPFFITNIMAVICKESCNEDVIGALLNVFVWIGYLSSAVNPLVYTLFNKTYRSAFSRYIQCQYKENKKPLQLILVNTIPALAYKSSQL<br>QMGQKKNSKQDAKTTDNDCSMVALGKQHSEEASKDNSDGVNEKVSCVGSSGGGGSGGGGSSGVTGYRLFEEIL						
**HSV-TK-3xHA-CB$_1$R-LgBiT**	pBiT1.1-C [TK/LgBiT]	FW	TCTGCCGAGGCTCTGGG CTCGAGCGGTGGT	pcDNA3.1(+)-3xHA-CB$_1$R	FW	GCTCAGGGGAATTCTGGTACC ACCATGTACCC
		RV	GTACATGGTGGTACCAG AATTCCCCTGAGCTCC		RV	ACCACCGCTCGAGCCCAGAG CCTCGGCAGA
MYPYDVPDYAYPYDVPDYAYPYDVPDYADKSILDGLADTTFRTITTDLLYVGSNDIQYEDIKGDMASKLGYFPQKFPLTSFRGSPFQE<br>KMTAGDNPQLVPADQVNITEFYNKSLSSFKENEENIQCGENFMDIECFMVLNPSQQLAIAVLSLTLGTFTVLENLLVLCVILHSRSLRC<br>RPSYHFIGSLAVADLLGSVIFVYSFIDFHVFHRKDSRNVFLFKLGGVTASFTASVGSLFLTAIDRYISIHRPLAYKRIVTRPKAVVAFCLM<br>WTIAIVIAVLPLLGWNCEKLQSVCSDIFPHIDETYLMFWIGVTSVLLLFIVYAYMYILWKAHSHAVRMIQRGTQKSIIIHTSEDGKVQVTRP<br>DQARMDIRLAKTLVLILVVLIICWGPLLAIMVYDVFGKMNKLIKTVFAFCSMLCLLNSTVNPIIYALRSKDLRHAFRSMFPSCEGTAQPLD<br>NSMGDSDCLHKHANNAASVHRAAESCIKSTVKIAKVTMSVSTDTSAEALGSSGGGGSGGGGSSGVFTLEDFVGDWEQTAAYNLDQ<br>VLEQGGVSSLLQNLAVSVTPIQRIVRSGENALKIDIHVIIPYEGLSADQMAQIEEVFKVVYPVDDHHFKVILPYGTLVIDGVTPNMLNYFG<br>RPYEGIAVFDGKKITVTGTLWNGNKIIDERLITPDGSMLFRVTINS						
**HSV-TK-3xHA-CB$_1$R-SmBiT**	pBiT2.1-C [TK/SmBiT]	FW	TCTGCCGAGGCTCTGGG CTCGAGCGGTGGT	pcDNA3.1(+)-3xHA-CB$_1$R	FW	GCTCAGGGGAATTCTGGTACC ACCATGTACCC
		RV	GTACATGGTGGTACCAG AATTCCCCTGAGCTCC		RV	ACCACCGCTCGAGCCCAGAG CCTCGGCAGA
MYPYDVPDYAYPYDVPDYAYPYDVPDYADKSILDGLADTTFRTITTDLLYVGSNDIQYEDIKGDMASKLGYFPQKFPLTSFRGSPFQE<br>KMTAGDNPQLVPADQVNITEFYNKSLSSFKENEENIQCGENFMDIECFMVLNPSQQLAIAVLSLTLGTFTVLENLLVLCVILHSRSLRC<br>RPSYHFIGSLAVADLLGSVIFVYSFIDFHVFHRKDSRNVFLFKLGGVTASFTASVGSLFLTAIDRYISIHRPLAYKRIVTRPKAVVAFCLM<br>WTIAIVIAVLPLLGWNCEKLQSVCSDIFPHIDETYLMFWIGVTSVLLLFIVYAYMYILWKAHSHAVRMIQRGTQKSIIIHTSEDGKVQVTRP<br>DQARMDIRLAKTLVLILVVLIICWGPLLAIMVYDVFGKMNKLIKTVFAFCSMLCLLNSTVNPIIYALRSKDLRHAFRSMFPSCEGTAQPLD<br>NSMGDSDCLHKHANNAASVHRAAESCIKSTVKIAKVTMSVSTDTSAEALGSSGGGGSGGGGSSGVTGYRLFEEIL						

**CMV-3xHA-5-HT$_{2A}$R-LgBiT**	pcDNA3.1(+)-3xHA-5-HT$_{2A}$R	FW	GTAACCATCAACAGCTG ACTCGAGTCTAGAGGG	pBiT1.1-C [TK/LgBiT]	FW	AAGGTGAGCTGTGTGGGCTC GAGCGGTGGT
		RV	ACCACCGCTCGAGCCCA CACAGCTCACCTTTTC		RV	TCTAGACTCGAGTCAGCTGTT GATGGTTACTCGG
MYPYDVPDYAYPYDVPDYAYPYDVPDYADDILCEENTSLSSTTNSLMQLNDDTRLYSNDFNSGEANTSDAFNWTVDSENRTNLSCEGCLSPSCLSLLHLQEKNWSALLTAVVIILTIAGNILVIMAVSLEKKLQNATNYFLMSLAIADMLLGFLVMPVSMLTILYGYRWPLPSKLCAVWIYLDVLFSTASIMHLCAISLDRYVAIQNPIHHSRFNSRTKAFLKIIAVWTISVGISMPIPVFGLQDDSKVFKEGSCLLADDNFVLIGSFVSFFIPLTIMVITYFLTIKSLQKEATLCVSDLGTRAKLASFSFLPQSSLSSEKLFQRSIHREPGSYTGRRTMQSISNEQKACKVLGIVFFLFVVMWCPFFITNIMAVICKESCNEDVIGALLNVFVWIGYLSSAVNPLVYTLFNKTYRSAFSRYIQCQYKENKKPLQLILVNTIPALAYKSSQLQMGQKKNSKQDAKTTDNDCSMVALGKQHSEEASKDNSDGVNEKVSCVGSSGGGGSGGGGSSGVFTLEDFVGDWEQTAAYNLDQVLEQGGVSSLLQNLAVSVTPIQRIVRSGENALKIDIHVIIPYEGLSADQMAQIEEVFKVVYPVDDHHFKVILPYGTLVIDGVTPNMLNYFGRPYEGIAVFDGKKITVTGTLWNGNKIIDERLITPDGSMLFRVTINS						
**CMV-3xHA-5-HT$_{2A}$R-SmBiT**	pcDNA3.1(+)-3xHA-5-HT$_{2A}$R	FW	TTCGAGGAGATTCTGTG ACTCGAGTCTAGAGGG	pBiT2.1-C [TK/SmBiT]	FW	AAGGTGAGCTGTGTGGGCTC GAGCGGTGGT
		RV	ACCACCGCTCGAGCCCA CACAGCTCACCTTTTC		RV	TCTAGACTCGAGTCACAGAAT CTCCTCGAACAGC
MYPYDVPDYAYPYDVPDYAYPYDVPDYADDILCEENTSLSSTTNSLMQLNDDTRLYSNDFNSGEANTSDAFNWTVDSENRTNLSCEGCLSPSCLSLLHLQEKNWSALLTAVVIILTIAGNILVIMAVSLEKKLQNATNYFLMSLAIADMLLGFLVMPVSMLTILYGYRWPLPSKLCAVWIYLDVLFSTASIMHLCAISLDRYVAIQNPIHHSRFNSRTKAFLKIIAVWTISVGISMPIPVFGLQDDSKVFKEGSCLLADDNFVLIGSFVSFFIPLTIMVITYFLTIKSLQKEATLCVSDLGTRAKLASFSFLPQSSLSSEKLFQRSIHREPGSYTGRRTMQSISNEQKACKVLGIVFFLFVVMWCPFFITNIMAVICKESCNEDVIGALLNVFVWIGYLSSAVNPLVYTLFNKTYRSAFSRYIQCQYKENKKPLQLILVNTIPALAYKSSQLQMGQKKNSKQDAKTTDNDCSMVALGKQHSEEASKDNSDGVNEKVSCVGSSGGGGSGGGGSSGVTGYRLFEEIL						
**CMV-3xHA-CB$_1$R-LgBiT**	pcDNA3.1(+)-3xHA-CB$_1$R	FW	GTAACCATCAACAGCTG ACTCGAGTCTAGAGGG	pBiT1.1-C [TK/LgBiT]	FW	TCTGCCGAGGCTCTGGGCTC GAGCGGTGGT
		RV	ACCACCGCTCGAGCCCA GAGCCTCGGCAGA		RV	TCTAGACTCGAGTCAGCTGTT GATGGTTACTCGG
MYPYDVPDYAYPYDVPDYAYPYDVPDYADKSILDGLADTTFRTITTDLLYVGSNDIQYEDIKGDMASKLGYFPQKFPLTSFRGSPFQEKMTAGDNPQLVPADQVNITEFYNKSLSSFKENEENIQCGENFMDIECFMVLNPSQQLAIAVLSLTLGTFTVLENLLVLCVILHSRSLRCRPSYHFIGSLAVADLLGSVIFVYSFIDFHVFHRKDSRNVFLFKLGGVTASFTASVGSLFLTAIDRYISIHRPLAYKRIVTRPKAVVAFCLMWTIAIVIAVLPLLGWNCEKLQSVCSDIFPHIDETYLMFWIGVTSVLLLFIVYAYMYILWKAHSHAVRMIQRGTQKSIIIHTSEDGKVQVTRPDQARMDIRLAKTLVLILVVLIICWGPLLAIMVYDVFGKMNKLIKTVFAFCSMLCLLNSTVNPIIYALRSKDLRHAFRSMFPSCEGTAQPLDNSMGDSDCLHKHANNAASVHRAAESCIKSTVKIAKVTMSVSTDTSAEALGSSGGGGSGGGGSSGVFTLEDFVGDWEQTAAYNLDQVLEQGGVSSLLQNLAVSVTPIQRIVRSGENALKIDIHVIIPYEGLSADQMAQIEEVFKVVYPVDDHHFKVILPYGTLVIDGVTPNMLNYFGRPYEGIAVFDGKKITVTGTLWNGNKIIDERLITPDGSMLFRVTINS						
**CMV-3xHA-CB$_1$R-SmBiT**	pcDNA3.1(+)-3xHA-CB$_1$R	FW	TTCGAGGAGATTCTGTG ACTCGAGTCTAGAGGG	pBiT2.1-C [TK/SmBiT]	FW	TCTGCCGAGGCTCTGGGCTC GAGCGGTGGT
		RV	ACCACCGCTCGAGCCCA GAGCCTCGGCAGA		RV	TCTAGACTCGAGTCACAGAAT CTCCTCGAACAGC
MYPYDVPDYAYPYDVPDYAYPYDVPDYADKSILDGLADTTFRTITTDLLYVGSNDIQYEDIKGDMASKLGYFPQKFPLTSFRGSPFQEKMTAGDNPQLVPADQVNITEFYNKSLSSFKENEENIQCGENFMDIECFMVLNPSQQLAIAVLSLTLGTFTVLENLLVLCVILHSRSLRCRPSYHFIGSLAVADLLGSVIFVYSFIDFHVFHRKDSRNVFLFKLGGVTASFTASVGSLFLTAIDRYISIHRPLAYKRIVTRPKAVVAFCLMWTIAIVIAVLPLLGWNCEKLQSVCSDIFPHIDETYLMFWIGVTSVLLLFIVYAYMYILWKAHSHAVRMIQRGTQKSIIIHTSEDGKVQVTRPDQARMDIRLAKTLVLILVVLIICWGPLLAIMVYDVFGKMNKLIKTVFAFCSMLCLLNSTVNPIIYALRSKDLRHAFRSMFPSCEGTAQPLDNSMGDSDCLHKHANNAASVHRAAESCIKSTVKIAKVTMSVSTDTSAEALGSSGGGGSGGGGSSGVTGYRLFEEIL						
**CMV-LgBiT-5-HT$_{2A}$R**	pcDNA3.1(+)-3xHA-5-HT$_{2A}$R	FW	GGAGGCTCGAGCGGTG ATATTCTTTGTGAAGAAA ATACTT	pBiT1.1-N [TK/LgBiT]	FW	AAGCTTGGTACCACCATGGTC TTCACACTCGAAG
		RV	GAGTGTGAAGACCATGG		RV	TTCACAAAGAATATCACCGCT

<table>
<tr><td></td><td></td><td></td><td>TGGTACCAAGCTTAAG</td><td></td><td></td><td>CGAGCCTCC</td></tr>
<tr><td colspan="7">MVFTLEDFVGDWEQTAAYNLDQVLEQGGVSSLLQNLAVSVTPIQRIVRSGENALKIDIHVIIPYEGLSADQMAQIEEVFKVVYPVDDHHFKVILPYGTLVIDGVTPNMLNYFGRPYEGIAVFDGKKITVTGTLWNGNKIIDERLITPDGSMLFRVTINSGSSGGGGSGGGGSSGDILCEENTSLSSTTNSLMQLNDDTRLYSNDFNSGEANTSDAFNWTVDSENRTNLSCEGCLSPSCLSLLHLQEKNWSALLTAVVIILTIAGNILVIMAVSLEKKLQNATNYFLMSLAIADMLLGFLVMPVSMLTILYGYRWPLPSKLCAVWIYLDVLFSTASIMHLCAISLDRYVAIQNPIHHSRFNSRTKAFLKIIAVWTISVGISMPIPVFGLQDDSKVFKEGSCLLADDNFVLIGSFVSFFIPLTIMVITYFLTIKSLQKEATLCVSDLGTRAKLASFSFLPQSSLSSEKLFQRSIHREPGSYTGRRTMQSISNEQKACKVLGIVFFLFVVMWCPFFITNIMAVICKESCNEDVIGALLNVFVWIGYLSSAVNPLVYTLFNKTYRSAFSRYIQCQYKENKKPLQLILVNTIPALAYKSSQLQMGQKKNSKQDAKTTDNDCSMVALGKQHSEEASKDNSDGVNEKVSCV</td></tr>
<tr><td rowspan="2">CMV-SmBiT-5-HT$_{2A}$R</td><td rowspan="2">pcDNA3.1(+)-3xHA-5-HT$_{2A}$R</td><td>FW</td><td>GGAGGCTCGAGCGGTGATATTCTTTGTGAAGAAATACTT</td><td rowspan="2">pBiT2.1-N [TK/SmBiT]</td><td>FW</td><td>AAGCTTGGTACCACCATGGTGACCGGCTACC</td></tr>
<tr><td>RV</td><td>GTAGCCGGTCACCATGGTGGTACCAAGCTTAAG</td><td>RV</td><td>TTCACAAAGAATATCACCGCTCGAGCCTCC</td></tr>
<tr><td colspan="7">MVTGYRLFEEILGSSGGGGSGGGGSSGDILCEENTSLSSTTNSLMQLNDDTRLYSNDFNSGEANTSDAFNWTVDSENRTNLSCEGCLSPSCLSLLHLQEKNWSALLTAVVIILTIAGNILVIMAVSLEKKLQNATNYFLMSLAIADMLLGFLVMPVSMLTILYGYRWPLPSKLCAVWIYLDVLFSTASIMHLCAISLDRYVAIQNPIHHSRFNSRTKAFLKIIAVWTISVGISMPIPVFGLQDDSKVFKEGSCLLADDNFVLIGSFVSFFIPLTIMVITYFLTIKSLQKEATLCVSDLGTRAKLASFSFLPQSSLSSEKLFQRSIHREPGSYTGRRTMQSISNEQKACKVLGIVFFLFVVMWCPFFITNIMAVICKESCNEDVIGALLNVFVWIGYLSSAVNPLVYTLFNKTYRSAFSRYIQCQYKENKKPLQLILVNTIPALAYKSSQLQMGQKKNSKQDAKTTDNDCSMVALGKQHSEEASKDNSDGVNEKVSCV</td></tr>
<tr><td rowspan="2">CMV-LgBiT-CB$_1$R</td><td rowspan="2">pcDNA3.1(+)-3xHA-CB$_1$R</td><td>FW</td><td>GGAGGCTCGAGCGGTAAGTCGATCCTAGATGGCC</td><td rowspan="2">pBiT1.1-N [TK/LgBiT]</td><td>FW</td><td>AAGCTTGGTACCACCATGGTCTTCACACTCGAAG</td></tr>
<tr><td>RV</td><td>GAGTGTGAAGACCATGGTGGTACCAAGCTTAAGT</td><td>RV</td><td>ATCTAGGATCGACTTACCGCTCGAGCCTCC</td></tr>
<tr><td colspan="7">MVFTLEDFVGDWEQTAAYNLDQVLEQGGVSSLLQNLAVSVTPIQRIVRSGENALKIDIHVIIPYEGLSADQMAQIEEVFKVVYPVDDHHFKVILPYGTLVIDGVTPNMLNYFGRPYEGIAVFDGKKITVTGTLWNGNKIIDERLITPDGSMLFRVTINSGSSGGGGSGGGGSSGKSILDGLADTTFRTITTDLLYVGSNDIQYEDIKGDMASKLGYFPQKFPLTSFRGSPFQEKMTAGDNPQLVPADQVNITEFYNKSLSSFKENEENIQCGENFMDIECFMVLNPSQQLAIAVLSLTLGTFTVLENLLVLCVILHSRSLRCRPSYHFIGSLAVADLLGSVIFVYSFIDFHVFHRKDSRNVFLFKLGGVTASFTASVGSLFLTAIDRYISIHRPLAYKRIVTRPKAVVAFCLMWTIAIVIAVLPLLGWNCEKLQSVCSDIFPHIDETYLMFWIGVTSVLLLFIVYAYMYILWKAHSHAVRMIQRGTQKSIIIHTSEDGKVQVTRPDQARMDIRLAKTLVLILVVLIICWGPLLAIMVYDVFGKMNKLIKTVFAFCSMLCLLNSTVNPIIYALRSKDLRHAFRSMFPSCEGTAQPLDNSMGDSDCLHKHANNAASVHRAAESCIKSTVKIAKVTMSVSTDTSAEAL</td></tr>
<tr><td rowspan="2">CMV-SmBiT-CB$_1$R</td><td rowspan="2">pcDNA3.1(+)-3xHA-CB$_1$R</td><td>FW</td><td>GGAGGCTCGAGCGGTAAGTCGATCCTAGATGGCC</td><td rowspan="2">pBiT2.1-N [TK/SmBiT]</td><td>FW</td><td>AAGCTTGGTACCACCATGGTGACCGGCTACC</td></tr>
<tr><td>RV</td><td>GTAGCCGGTCACCATGGTGGTACCAAGCTTAAGT</td><td>RV</td><td>ATCTAGGATCGACTTACCGCTCGAGCCTCC</td></tr>
<tr><td colspan="7">MVTGYRLFEEILGSSGGGGSGGGGSSGKSILDGLADTTFRTITTDLLYVGSNDIQYEDIKGDMASKLGYFPQKFPLTSFRGSPFQEKMTAGDNPQLVPADQVNITEFYNKSLSSFKENEENIQCGENFMDIECFMVLNPSQQLAIAVLSLTLGTFTVLENLLVLCVILHSRSLRCRPSYHFIGSLAVADLLGSVIFVYSFIDFHVFHRKDSRNVFLFKLGGVTASFTASVGSLFLTAIDRYISIHRPLAYKRIVTRPKAVVAFCLMWTIAIVIAVLPLLGWNCEKLQSVCSDIFPHIDETYLMFWIGVTSVLLLFIVYAYMYILWKAHSHAVRMIQRGTQKSIIIHTSEDGKVQVTRPDQARMDIRLAKTLVLILVVLIICWGPLLAIMVYDVFGKMNKLIKTVFAFCSMLCLLNSTVNPIIYALRSKDLRHAFRSMFPSCEGTAQPLDNSMGDSDCLHKHANNAASVHRAAESCIKSTVKIAKVTMSVSTDTSAEAL</td></tr>
<tr><td rowspan="2">CMV-3xHA-5-HT$_{2A}$R-VN173</td><td rowspan="2">CMV-3xHA-5-HT$_{2A}$R-SmBiT</td><td>FW</td><td>CACAACATCGAGTAGCTCGAGTCTAGAGGGCC</td><td rowspan="2">pBiFC-bJunVN173</td><td>FW</td><td>GGAGGGTCGTCAGGTATGGTGAGCAAGGGCG</td></tr>
<tr><td>RV</td><td>GCCCTTGCTCACCATACCTGACGACCCTCC</td><td>RV</td><td>CCCTCTAGACTCGAGCTACTCGATGTTGTGGCG</td></tr>
<tr><td colspan="7">MYPYDVPDYAYPYDVPDYAYPYDVPDYADILCEENTSLSSTTNSLMQLNDDTRLYSNDFNSGEANTSDAFNWTVDSENRTNLSCEGCLSPSCLSLLHLQEKNWSALLTAVVIILTIAGNILVIMAVSLEKKLQNATNYFLMSLAIADMLLGFLVMPVSMLTILYGYRWPLPSKLCAVWIYLDVLFSTASIMHLCAISLDRYVAIQNPIHHSRFNSRTKAFLKIIAVWTISVGISMPIPVFGLQDDSKVFKEGSCLLADDNFVLIGSFVSFFIPLTIMVITYFLTIKSLQKEATLCVSDLGTRAKLASFSFLPQSSLSSEKLFQRSIHREPGSYTGRRTMQSISNEQKACKVLGIVFFLFV</td></tr>
</table>

VMWCPFFITNIMAVICKESCNEDVIGALLNVFVWIGYLSSAVNPLVYTLFNKTYRSAFSRYIQCQYKENKKPLQLILVNTIPALAYKSSQLQMGQKKNSKQDAKTTDNDCSMVALGKQHSEEASKDNSDGVNEKVSCVGSSGGGGSGGGGSSGMVSKGEELFTGVVPILVELDGDVNGHKFSVSGEGEGDATYGKLTLKLICTTGKLPVPWPTLVTTLGYGLQCFARYPDHMKQHDFFKSAMPEGYVQERTIFFKDDGNYKTRAEVKFEGDTLVNRIELKGIDFKEDGNILGHKLEYNYNSHNVYITADKQKNGIKANFKIRHNIE						
**CMV-3xHA-5-HT$_{2A}$R-VC155**	CMV-3xHA-5-HT$_{2A}$R-SmBiT	FW	GAGCTGTACAAGTAACTCGAGTCTAGAGGGCC	pBiFC-bFosVC155	FW	GGAGGGTCGTCAGGTGACAAGCAGAAGAACGGC
		RV	GTTCTTCTGCTTGTCACCTGACGACCCTCC		RV	CCCTCTAGACTCGAGTTACTTGTACAGCTCGTCC
MYPYDVPDYAYPYDVPDYAYPYDVPDYADDILCEENTSLSSTTNSLMQLNDDTRLYSNDFNSGEANTSDAFNWTVDSENRTNLSCEGCLSPSCLSLLHLQEKNWSALLTAVVIILTIAGNILVIMAVSLEKKLQNATNYFLMSLAIADMLLGFLVMPVSMLTILYGYRWPLPSKLCAVWIYLDVLFSTASIMHLCAISLDRYVAIQNPIHHSRFNSRTKAFLKIIAVWTISVGISMPIPVFGLQDDSKVFKEGSCLLADDNFVLIGSFVSFFIPLTIMVITYFLTIKSLQKEATLCVSDLGTRAKLASFSFLPQSSLSSEKLFQRSIHREPGSYTGRRTMQSISNEQKACKVLGIVFFLFVVMWCPFFITNIMAVICKESCNEDVIGALLNVFVWIGYLSSAVNPLVYTLFNKTYRSAFSRYIQCQYKENKKPLQLILVNTIPALAYKSSQLQMGQKKNSKQDAKTTDNDCSMVALGKQHSEEASKDNSDGVNEKVSCVGSSGGGGSGGGGSSGDKQKNGIKANFKIRHNIEDGGVQLADHYQQNTPIGDGPVLLPDNHYLSYQSKLSKDPNEKRDHMVLLEFVTAAGITLGMDELYK						
**CMV-3xHA-CB$_1$R-VN173**	CMV-3xHA-CB$_1$R-SmBiT	FW	CACAACATCGAGTAGCTCGAGTCTAGAGGGCC	pBiFC-bJunVN173	FW	GGAGGGTCGTCAGGTATGGTGAGCAAGGGCG
		RV	GCCCTTGCTCACCATACCTGACGACCCTCC		RV	CCCTCTAGACTCGAGCTACTCGATGTTGTGGCG
MYPYDVPDYAYPYDVPDYAYPYDVPDYADKSILDGLADTTFRTITTDLLYVGSNDIQYEDIKGDMASKLGYFPQKFPLTSFRGSPFQEKMTAGDNPQLVPADQVNITEFYNKSLSSFKENEENIQCGENFMDIECFMVLNPSQQLAIAVLSLTLGTFTVLENLLVLCVILHSRSLRCRPSYHFIGSLAVADLLGSVIFVYSFIDFHVFHRKDSRNVFLFKLGGVTASFTASVGSLFLTAIDRYISIHRPLAYKRIVTRPKAVVAFCLMWTIAIVIAVLPLLGWNCEKLQSVCSDIFPHIDETYLMFWIGVTSVLLLFIVYAYMYILWKAHSHAVRMIQRGTQKSIIIHTSEDGKVQVTRPDQARMDIRLAKTLVLILVVLIICWGPLLAIMVYDVFGKMNKLIKTVFAFCSMLCLLNSTVNPIIYALRSKDLRHAFRSMFPSCEGTAQPLDNSMGDSDCLHKHANNAASVHRAAESCIKSTVKIAKVTMSVSTDTSAEALGSSGGGGSGGGGSSGMVSKGEELFTGVVPILVELDGDVNGHKFSVSGEGEGDATYGKLTLKLICTTGKLPVPWPTLVTTLGYGLQCFARYPDHMKQHDFFKSAMPEGYVQERTIFFKDDGNYKTRAEVKFEGDTLVNRIELKGIDFKEDGNILGHKLEYNYNSHNVYITADKQKNGIKANFKIRHNIE						
**CMV-3xHA-CB$_1$R-VC155**	CMV-3xHA-CB$_1$R-SmBiT	FW	GAGCTGTACAAGTAACTCGAGTCTAGAGGGCC	pBiFC-bFosVC155	FW	GGAGGGTCGTCAGGTGACAAGCAGAAGAACGGC
		RV	GTTCTTCTGCTTGTCACCTGACGACCCTCC		RV	CCCTCTAGACTCGAGTTACTTGTACAGCTCGTCC
MYPYDVPDYAYPYDVPDYAYPYDVPDYADKSILDGLADTTFRTITTDLLYVGSNDIQYEDIKGDMASKLGYFPQKFPLTSFRGSPFQEKMTAGDNPQLVPADQVNITEFYNKSLSSFKENEENIQCGENFMDIECFMVLNPSQQLAIAVLSLTLGTFTVLENLLVLCVILHSRSLRCRPSYHFIGSLAVADLLGSVIFVYSFIDFHVFHRKDSRNVFLFKLGGVTASFTASVGSLFLTAIDRYISIHRPLAYKRIVTRPKAVVAFCLMWTIAIVIAVLPLLGWNCEKLQSVCSDIFPHIDETYLMFWIGVTSVLLLFIVYAYMYILWKAHSHAVRMIQRGTQKSIIIHTSEDGKVQVTRPDQARMDIRLAKTLVLILVVLIICWGPLLAIMVYDVFGKMNKLIKTVFAFCSMLCLLNSTVNPIIYALRSKDLRHAFRSMFPSCEGTAQPLDNSMGDSDCLHKHANNAASVHRAAESCIKSTVKIAKVTMSVSTDTSAEALGSSGGGGSGGGGSSGDKQKNGIKANFKIRHNIEDGGVQLADHYQQNTPIGDGPVLLPDNHYLSYQSKLSKDPNEKRDHMVLLEFVTAAGITLGMDELYK						

Primer sequences for Gibson cloning. The colour code corresponds to the translated sequences of: HA-Tag, 5-HT$_{2A}$R/CB$_1$R, G/S flexible Linker, LgBiT, SmBiT, VN173 Venus YFP and CV155 Venus YFP.
